# Association between glucosamine use and albuminuria in the UK: a cohort and Mendelian randomisation study

**DOI:** 10.1136/bmjopen-2024-096344

**Published:** 2025-11-21

**Authors:** Samantha JL Hayward, Andrei Constantinescu, Emma Hazelwood, Matthew J Butler, Emma E Vincent, Simon C Satchell

**Affiliations:** 1 Bristol Renal, Translational Health Sciences, Bristol Medical School, University of Bristol, Bristol, UK; 2 MRC-Integrative Epidemiology Unit, Population Health Sciences, Bristol Medical School, University of Bristol, Bristol, UK

**Keywords:** Mendelian Randomization Analysis, NEPHROLOGY, Chronic renal failure, Drug Therapy, EPIDEMIOLOGY

## Abstract

**Abstract:**

**Background:**

Glucosamine is a commonly used ‘over the counter’ dietary supplement. Previous research has identified an association between glucosamine use and several positive health outcomes. However, a plausible biological mechanism for these associations has not yet been identified, meaning the causality of these relationships remains unclear. A protective effect of glucosamine on the vascular endothelium has been suggested as one such possible mechanism. Albuminuria is an early marker of endothelial dysfunction within the kidney and is associated with progression of kidney disease and adverse cardiovascular outcomes. In order to provide insights into the potential biological mechanisms underlying a protective association of glucosamine use with health outcomes, we evaluated evidence for an association between glucosamine use and albuminuria in UK Biobank (N=436 200).

**Methods:**

Univariable and multivariable ordinal logistic regression were performed to evaluate evidence for an association between self-reported glucosamine use and albuminuria (measured as urine albumin creatinine ratio (uACR) categories). As a secondary analysis, we performed Mendelian randomisation (MR) to demonstrate the difficulties in inferring causality in this relationship using currently available data, using summary genetic data from UK Biobank and CDKGen (N=67 452).

**Results:**

We found that people who used glucosamine were more likely to be in a lower uACR group (OR 0.81, 95% CI 0.80 to 0.83, p<2.2×10^−16^). This association was robust to sensitivity analyses and was maintained after adjustment for age, sex and measures of obesity. In our MR analysis, we found little evidence for an association of genetically proxied glucosamine use on albuminuria (change in log uACR (mg/g) per SD change in genetic liability=1.11, 95% CI −3.01 to 5.23, p=0.60).

**Conclusions:**

We found that detectable albuminuria was common in UK Biobank participants and we are the first to show that use of glucosamine supplements was associated with lower levels. Though this fits with a plausible biological role of the vascular endothelium in a potential protective effect of glucosamine use on many health outcomes, whether this relationship is causal or confounded remains unclear. We further discuss the inherent difficulties in using genetic instruments to proxy supplement use in MR analyses and highlight the need for a genome-wide association study of measured circulating glucosamine levels.

**STRENGTHS AND LIMITATIONS OF THIS STUDY**

- The strengths of our study include the large sample size, use of clinically relevant urine albumin creatinine ratio categories and consistent findings across all subgroup and sensitivity analyses.
- Our findings may be influenced by residual or unmeasured confounding or participation bias.
- Glucosamine use was self-reported and we do not have details of the patterns of use.
- As discussed throughout the manuscript, genetic instruments proxying supplement use for Mendelian randomisation analyses likely suffer from horizontal pleiotropy.

## Introduction

 Glucosamine is required for the production of glycosylated proteins and lipids.^1^ It is commonly used as an ‘over the counter’ dietary supplement, particularly as a complementary treatment for osteoarthritis. Over the last decade, several observational studies have found an association between glucosamine use and a wide range of positive health outcomes. For example, people who use glucosamine have a decreased incidence of vascular dementia, stroke and cardiovascular disease, as well as lower all-cause mortality.^2^,^4^ However, a plausible biological mechanism for these observed findings is lacking, leading to scepticism about whether these associations are likely to be causal. Instead, given people who take glucosamine may have other positive health behaviours, an alternative explanation is that the observed relationships between glucosamine use and positive health outcomes may be a result of unmeasured or residual confounding.^5^

A protective effect of glucosamine on the vascular endothelium has been suggested as a possible mechanism explaining the positive health outcomes associated with glucosamine use. Albuminuria can develop as a result of endothelial dysfunction within the kidney.^6 7^ In clinical practice, microalbuminuria is used as an early marker of kidney dysfunction and higher levels are a risk factor for disease progression and the development of end-stage kidney disease.^8^ Albuminuria is also a risk factor for cardiovascular outcomes, suggesting that vascular endothelial health in the kidney is mirrored by other organs.^8 9^ As glucosamine use has been associated with lower vascular health-related outcomes across a variety of organs, a direct protective effect on the vascular endothelium throughout the body represents a plausible biological mechanism for a protective effect of glucosamine use on several downstream health outcomes. We explored this hypothesis by examining the association between glucosamine use and albuminuria in people recruited to the UK Biobank (UKBB).

Mendelian randomisation (MR), a type of instrumental variable analysis, uses genetic data to investigate the evidence for a causal relationship between an exposure and outcome.^10^ Previous MR analyses have suggested that glucosamine use is beneficial for a range of health-related outcomes.^4 11 12^ However, the use of genetic proxies of glucosamine use, rather than circulating glucosamine levels, in these analyses brings into question the reliability of these conclusions given the unknown causal pathway between genetic instruments and future supplement use.

The aims of this study are to:

1. Determine if there is an association between glucosamine use and albuminuria in UKBB.
2. Demonstrate the inherent issues in evaluating evidence for a causal effect of glucosamine use on albuminuria in an MR analysis using currently available data.

## Materials and methods

### Observational analysis using the UKBB

#### Study population

UKBB is a large prospective population-based cohort, which recruited 502 396 participants aged 40–70 from the UK between 1 June 2006 and 1 June 2010.^13^ Access to UKBB data was granted under application code 81499. All participants provided written informed consent at recruitment. Participants were excluded from our analysis if they had withdrawn their consent later in the study or had invalid study numbers (n=14 252), if they were missing urine albumin and creatinine measurements (n=14 391), or missing data on potential confounders (deprivation, body mass index (BMI), waist-to-hip ratio, smoking and blood pressure, n=34 531). Participants with liver disease were also excluded (n=2727), as this may affect their albumin metabolism. In addition, pregnant recruits (n=295) were excluded as albuminuria is likely to be driven by different underlying molecular processes in pregnancy.^14^ In total, 436 200 UKBB participants were included in our analysis.

#### Glucosamine and albuminuria data

UKBB participants attended 1 of 22 UKBB centres for their baseline assessment. During these visits, they underwent verbal interviews, assessments and completed touchscreen questionnaires. We created a composite glucosamine use variable from two of the UKBB data fields, both collected at the initial study visit. The first data field was from a verbal interview on prescription drug use conducted by a trained nurse during which participants were asked ‘Do you regularly take any prescription medications?’. The second data field was from a touchscreen questionnaire in which the recruits were asked ‘Do you take any of the following?’ and were supplied with a list of supplements, which included glucosamine. If glucosamine use was indicated in answer to either of these questions, the individual was classed as taking glucosamine. Urine albumin and urine creatinine values were collected at the UKBB baseline visit; these were converted to a urinary albumin creatinine ratio (uACR) and split into four categories (undetectable, < 3 mg/mmol, 3–30 mg/mmol, > 30 mg/mmol in keeping with Kidney Disease Improving Global Outcomes (KDIGO) albuminuria categories).^15^

#### Primary, sensitivity and subgroup analyses

Univariable and multivariable ordinal logistic regression were used to assess the relationship between glucosamine use and uACR categories. The multivariable model was adjusted for age, sex, BMI and waist-to-hip ratio. Additional sensitivity analyses were undertaken. First, as ordinal logistic regression assumes that any association between glucosamine use and uACR is constant across the albuminuria categories (proportional odds assumption), univariable linear regression was used to examine glucosamine use and uACR as a continuous variable; log transformation and rank inverse normalisation of the uACR data were used as the data were not normally distributed. Second, multivariable ordinal regression (assessing uACR categories) was used and adjusted for the same factors as the main analysis, plus smoking and blood pressure. Finally, subgroup analyses were performed examining whether different associations were seen in people with and without diabetes, as well as people with higher and lower levels of kidney function (classed as estimated glomerular filtration rate (eGFR) of above or below 60 mL/min/1.73 m^2^). People with missing diabetes (n=8, <1%) or eGFR (20 668 people, 4.7%) data were excluded from the respective subgroup analyses.

### Two-sample MR

**Figure 1:**
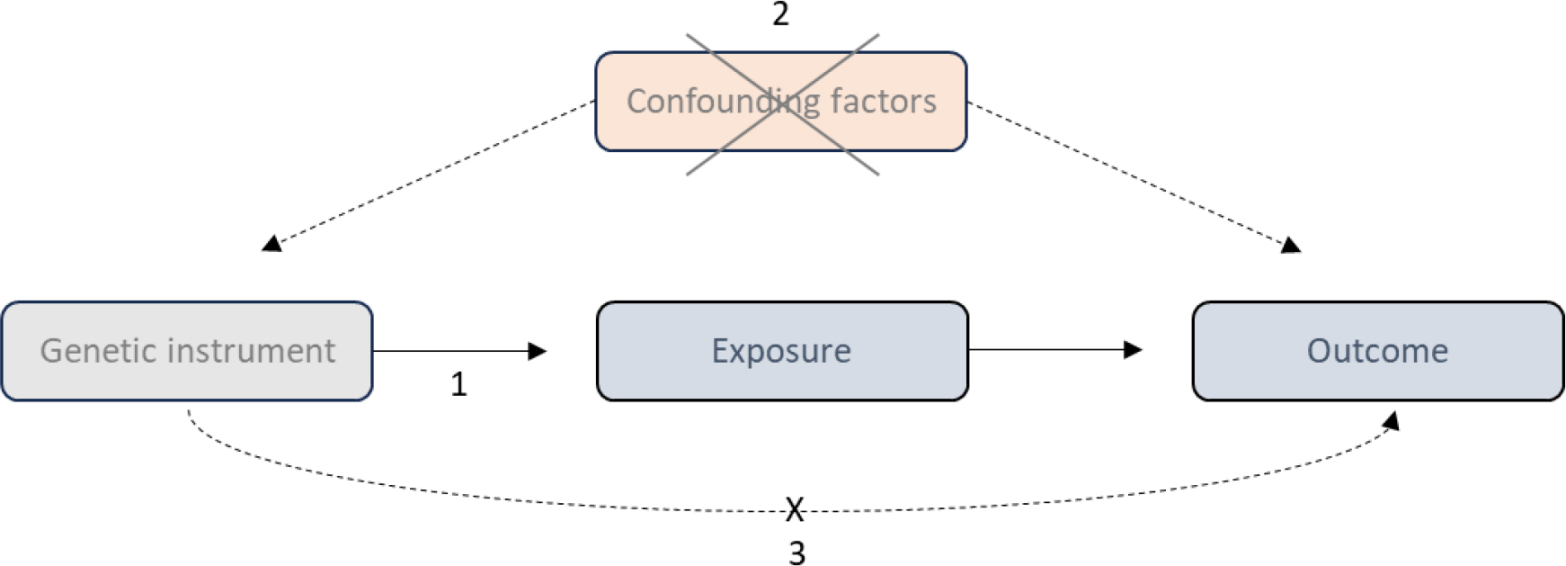
Directed acyclic graph demonstrating the core assumptions of Mendelian randomisation. 1. Relevance, 2. Independence, 3. Exclusion restriction.

MR has three core instrumental variable assumptions (illustrated in figure 1):

1. Relevance—The instrumental variables are strongly associated with the exposure.
2. Independence—The instrumental variables are not affected by confounding.
3. Exclusion restriction—There is no horizontal pleiotropy as the instrumental variable is independent of the outcome except via the exposure.

At present, there are no genome-wide association studies (GWAS) of circulating glucosamine levels, and without this, MR cannot robustly determine whether glucosamine has a causal effect on any disease. Instead, only the relationship between genetic predisposition to taking glucosamine supplements can be explored using MR. We performed a two-sample MR analysis, using summary-level data, to examine the evidence for a causal relationship between genetic predisposition to taking glucosamine supplements and uACR.

GWAS summary data were obtained from the MRC-IEU OpenGWAS data infrastructure.^16^ Summary data from a glucosamine use UKBB GWAS were used (Open GWAS ID: ukb-b-11535), single-nucleotide polymorphisms (SNPs) with a genome-wide significant association (p<5×10^−8^) were selected as instrumental variables (further details provided in online supplemental table 1). Linkage disequilibrium clumping of SNPs was performed to ensure their independence (r^2^ <0.001). The F-statistic was calculated to determine whether each instrument was strongly associated with glucosamine use, with an F-statistic greater than 10 taken to be indicative of minimal weak instrument bias, and R^2^ was calculated to determine the variance in glucosamine use explained by each SNP (online supplemental table 2). SNP-outcome associations were obtained from a microalbuminuria GWAS meta-analysis from the CKDGen consortium (n=67 452 participants); microalbuminuria was measured as log(uACR in mg/g).^17^ Both the glucosamine and microalbuminuria GWASs were performed in people of European ancestry from the general population. Both cohorts included people with and without diabetes and kidney disease. The glucosamine GWAS was adjusted for sex, genotyping array and 10 genetic principal components. The microalbuminuria GWAS meta-analysis was adjusted for age, study centre and 10 genetic principal components, as well as the study-specific covariates for each of the individual GWAS studies.^17^ There was low heterogeneity across the GWAS studies included in the CKDGen meta-analysis.^17^ All original GWAS studies received appropriate ethical approval.

Inverse-variance weighting (IVW) was used for the main effect estimate of genetically predicted glucosamine use on uACR. IVW assumes that all the SNPs used in the genetic instrument are valid.^18^ Therefore, the inclusion of pleiotropic variants in an IVW analysis can lead to biased causal effect estimates.^19^ To address this assumption and evaluate the possibility of horizontal pleiotropy, sensitivity analyses using MR-Egger, weighted median and mode were performed; these methods can offer more consistent causal effect estimates when the IV assumptions are violated but have different underlying assumptions. MR-Egger can give consistent estimates when 100% of the genetic variants are invalid IVs.^20^ However, MR-Egger requires that the pleiotropic effects of the IV SNPs on the outcome are independent of their associations with the exposure (Instrument Strength Independent of Direct Effects–InSIDE–assumption).^20^ Both weighted median and weighted mode approaches can produce more consistent causal estimates regardless of the type of horizontal pleiotropy (ie, the InSIDE assumption can be violated).^20^ Weighted median can provide consistent estimates when less than 50% of the IVs are invalid, whereas weighted mode can perform well in the context of 50%–100% invalid IVs.^20^ A weighted mode approach carries the additional assumption that the largest number of similar SNP-specific causal estimates comes from the valid IVs (Zero Modal Pleiotropy Assumption–ZEMPA).^20^ Alongside this, to assess for heterogeneity in the causal estimates for each SNP, Cochrane’s Q test was performed. All analyses were performed in R V.4.2.1, using the ‘TwoSampleMR’ R package.^21^

### Phenome-wide association analysis

In order to demonstrate the issues in using liability to glucosamine use, rather than measured glucosamine levels, as an exposure in MR analyses, we performed a phenome-wide association analysis. We used the IEU OpenGWAS PheWAS platform (available: https://gwas.mrcieu.ac.uk/phewas/, accessed on 4 November 2024) to evaluate the associations between the five genetic instruments employed to proxy glucosamine use in our MR analyses (as detailed in online supplemental table 1) and all 50 044 available outcomes. We used a p value of <9.99×10^−7^ (0.05/50 044 outcomes) as evidence for an association.

### Patient and public involvement

Patients and the public were not involved in the design, conduct, reporting or dissemination of this study.

## Results

### Cohort description

In the entire study cohort, detectable albuminuria was common (n=1 36 253, 31.2%), with 22 647 people (5.2%) having moderately or severely increased levels. Of the 436 200 UKBB participants, 85 143 people (19.5%) reported regular glucosamine use. A greater proportion of the glucosamine users were women (n=52 888, 62.1%) and had lower levels of deprivation (Townsend deprivation index quintile 5, n=12 639, 14.8%, compared with people who do not take glucosamine, n=74 596, 21.2%). Mean blood pressures for the two groups were similar (141/82 mm/Hg in glucosamine users vs 139/82 mm/Hg in non-users). The full characteristics of the UKBB study cohort are listed in table 1.

**Table 1:** Characteristics of the UK Biobank study cohort

Characteristic	Glucosamine non-user	Glucosamine user	Overall
Number of participants	351 057	85 143	436 200
Age			
Mean (SD)	55.9 (8.19)	59.1 (7.06)	56.5 (8.08)
Sex			
Female	183 112 (52.2%)	52 888 (62.1%)	236 000 (54.1%)
Diabetes	19 010 (5.4%)	3033 (3.6%)	22 043 (5.1%)
Kidney function			
eGFR <60 mL/min/1.73m^2^	7896 (2.2%)	1723 (2.0%)	9619 (2.2%)
Smoking			
Never	192 243 (54.8%)	46 984 (55.2%)	239 227 (54.8%)
Previous	119 184 (34.0%)	32 656 (38.4%)	151 840 (34.8%)
Current	39 630 (11.3%)	5503 (6.5%)	45 133 (10.3%)
Townsend deprivation index (quintiles)			
Q1	67 671 (19.3%)	19 583 (23.0%)	87 254 (20.0%)
Q2	68 321 (19.5%)	18 920 (22.2%)	87 241 (20.0%)
Q3	69 357 (19.8%)	17 875 (21.0%)	87 232 (20.0%)
Q4	71 112 (20.3%)	16 126 (18.9%)	87 238 (20.0%)
Q5	74 596 (21.2%)	12 639 (14.8%)	87 235 (20.0%)
Education			
Non-vocational qualification	23 272 (6.6%)	5280 (6.2%)	28 552 (6.5%)
Certificate of secondary education	20 567 (5.9%)	3574 (4.2%)	24 141 (5.5%)
General certificate of secondary education	73 814 (21.0%)	19 618 (23.0%)	93 432 (21.4%)
A-levels	38 645 (11.0%)	10 028 (11.8%)	48 673 (11.2%)
University degree	11 3728 (32.4%)	27 982 (32.9%)	141 710 (32.5%)
Other professional	17 289 (4.9%)	5204 (6.1%)	22 493 (5.2%)
None of the above	59 956 (17.1%)	12 716 (14.9%)	72 672 (16.7%)
Prefer not to answer or missing data	3786 (1.1%)	741 (1.0%)	4527 (1.0%)
BMI			
Mean (SD)	27.4 (4.77)	27.3 (4.59)	27.4 (4.73)
Waist-to-hip ratio			
Mean (SD)	0.874 (0.0902)	0.861 (0.0862)	0.872 (0.0896)
Systolic blood pressure			
Mean (SD)	139 (19.7)	141 (19.5)	140 (19.6)
Diastolic blood pressure			
Mean (SD)	82.3 (10.7)	82.0 (10.4)	82.2 (10.7)
uACR (mg/mmol)			
Undetectable: urine albumin 0	23 8467 (67.9%)	61 480 (72.2%)	299 947 (68.8%)
KDIGO: A1 Normal to mildly increased: uACR 0.1–3	93 677 (26.7%)	19 929 (23.4%)	113 606 (26.0%)
KDIGO: A2 Moderately increased: uACR 3–30	17 211 (4.9%)	3469 (4.1%)	20 680 (4.7%)
KDIGO: A3 Severely increased: uACR≥30	1702 (0.5%)	265 (0.3%)	1967 (0.5%)

Missing data: Diabetes status was missing for 8 people (<1%) and eGFR data were missing for 20 668 people (4.7%).

BMI, body mass index; eGFR, estimated glomerular filtration rate; KDIGO, Kidney Disease Improving Global Outcomes; uACR, urinary albumin creatinine ratio.

### Glucosamine use and uACR: observational analyses

**Figure 2:**
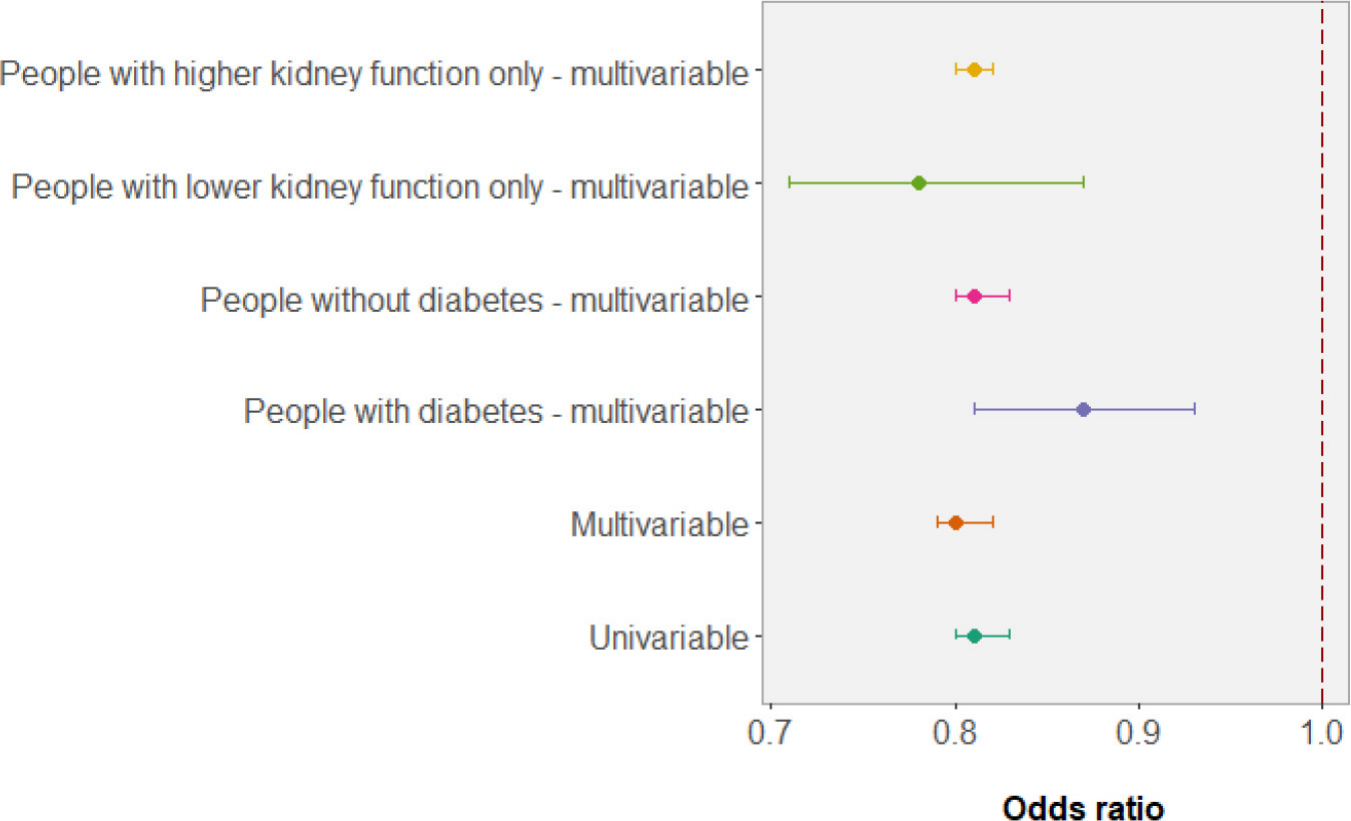
Association between glucosamine use and uACR categories. uACR, urine albumin creatinine ratio.

People who were taking glucosamine had 19% lower odds of being in a higher albuminuria category compared with those who were not taking glucosamine (OR 0.81, 95% CI 0.80 to 0.83, p<2.2×10^−16^, table 2 and figure 2). The association between glucosamine use and lower albuminuria was maintained after adjustment for age, sex and measures of obesity (table 2, OR 0.80, 95% CI 0.79 to 0.82, p<2.2×10^−16^).

**Table 2:** Observational UK Biobank results—association between glucosamine use and uACR

Model	OR	95% CIs	P value
Univariable ordinal logistic regression	0.81	0.80 to 0.83	<2.2×10^−16^
Multivariable ordinal logistic regression Adjusted for age, sex, body mass index and waist-to-hip ratio	0.80	0.79 to 0.82	<2.2×10^−16^

uACR, urinary albumin creatinine ratio.

### Sensitivity and subgroup analyses

Results were consistent in the sensitivity analyses, which examined uACR as a continuous variable (online supplemental table 3) and adjusted for smoking and blood pressure (OR 0.81, 95% CI 0.80 to 0.83, online supplemental table 4). In the subgroup analyses, the effect size was slightly attenuated in people with diabetes (OR 0.87, 95% CI 0.81 to 0.93, p=0.00017, online supplemental table 5 and figure 2) and marginally larger for people with lower levels of kidney function (OR 0.78, 95% CI 0.71 to 0.87, p=3.73×10^−6^, online supplemental table 5 and figure 2). Overall, these results show a strong independent association between glucosamine use and lower albuminuria in a large cohort of adults from the UK.

### MR analysis

Five SNPs were used to proxy glucosamine use (online supplemental table 2). SNP rs1466978 lies 229 base pairs upstream of DDB1 and CUL3-associated factor 4-like 1 gene (*DCAF4L1*) and rs34084719 is an intronic variant in component of oligomer golgi complex 5 (*COG5*). The other three SNPs used in the genetic instrument do not annotate to genes. There was little evidence for an association of genetically proxied glucosamine use on albuminuria across MR analyses (change in log uACR (mg/g) per SD change in genetic liability=1.11, 95% CI −3.01 to 5.23, p=0.60, table 3).

**Table 3:** Two-sample Mendelian randomisation results

**Method**	**Beta**	**95%** CI	**SE**	**P value**	**Q**	**Q P value**
IVW	1.11	−3.01 to 5.23	2.10	0.60	4.32	0.36
MR Egger	42.52	−7.79 to 92.84	25.67	0.20	1.59	0.66
Weighted median	0.30	−4.96 to 5.57	2.75	0.91		
Weighted mode	0.19	−7.10 to 7.48	3.72	0.96		

Betas represent change in log uACR (mg/g) per SD increase in genetic liability to glucosamine use.

IVW, inverse-variance weighting; MR, Mendelian randomisation; uACR, urine albumin creatinine ratio.

### Phenome-wide association analysis

In the phenome-wide association analysis, there was evidence (p<9.99×10^−7^) for an association of genetically proxied glucosamine uses genetic instruments and 211 outcomes, including taking fish oil supplements, alcohol consumption, height and adiposity measures (online supplemental tables 6–10).

## Discussion

In this analysis of 436 200 people from the UK, detectable albuminuria was common and glucosamine use was associated with lower albuminuria levels. People who used glucosamine had a 19% higher chance of being in a lower albuminuria group, as defined by the clinical KDIGO thresholds. Our findings were also consistent in people with diabetes and with lower levels of kidney function. By using these KDIGO albuminuria thresholds, our findings can be interpreted in relation to the known risks of albuminuria and progression to end stage kidney disease and adverse cardiovascular events, as well as being more easily translatable to a clinical setting. We highlight the need for further investigation into glucosamine as an albuminuria-lowering treatment.

Many studies have linked glucosamine use with a variety of better health outcomes, including lower incidence of cardiovascular disease, stroke, dementia, type 2 diabetes and heart failure, as well as cancer and all-cause mortality.^34 22^,^24^ Previous studies have speculated that these findings may be due to the anti-inflammatory effects of glucosamine. This anti-inflammatory hypothesis is supported by both in vitro work and human trial data. Glucosamine has been shown to reduce the synthesis of pro-inflammatory mediators in human osteoarthritic chondrocyte cell lines and rat articular chondrocyte cell lines.^25 26^ A randomised control trial of glucosamine demonstrated a reduction in the inflammatory marker C-Reactive Protein in the treatment group.^27^ In addition, proteomic analysis showed a reduction in cytokine interaction pathways in the glucosamine arm.^27^

Albuminuria occurs due to disruption of the glomerular filtration barrier, of which the endothelium is a major component. Glucosamine is a precursor of components of the endothelial glycocalyx, a carbohydrate-rich layer which lines the luminal surface of the vascular endothelium. The endothelial glycocalyx maintains capillary wall permeability, with some evidence that glycocalyx damage results in albuminuria and adverse renal and cardiovascular outcomes.^28^,^33^ Furthermore, dietary supplementation with glucosamine has been shown to improve a damaged glycocalyx in mice.^34^ Therefore, it is plausible that glucosamine is acting directly on the endothelial glycocalyx, providing a protective effect on the vascular endothelium throughout the body, resulting in positive health outcomes across many organs. This theory about glucosamine’s mechanism of action could also complement the anti-inflammatory hypothesis, as the endothelial glycocalyx helps regulate inflammatory cell extravasation and glycocalyx injury can be induced by inflammatory mediators or sepsis.^35^,^38^

Our study showed that the association between glucosamine use and lower albuminuria was maintained in people with diabetes and lower levels of kidney function, as well as those without these conditions. Medications which reduce albuminuria are recommended for people with chronic kidney disease, including diabetic kidney disease, as they slow the rate of GFR decline and progression to kidney failure.^39^ Glucosamine, therefore, has therapeutic potential in chronic kidney disease management, if the causality of this relationship can be determined. A significant proportion of the general population has detectable albuminuria (31.2% in this UKBB cohort) which is known to confer increased risk of all-cause and cardiovascular mortality.^40^ Albuminuria may identify people who have endothelial dysfunction and who are therefore most likely to benefit from glucosamine in terms of reduction of cardiovascular disease risk.^3^

**Figure 3:**
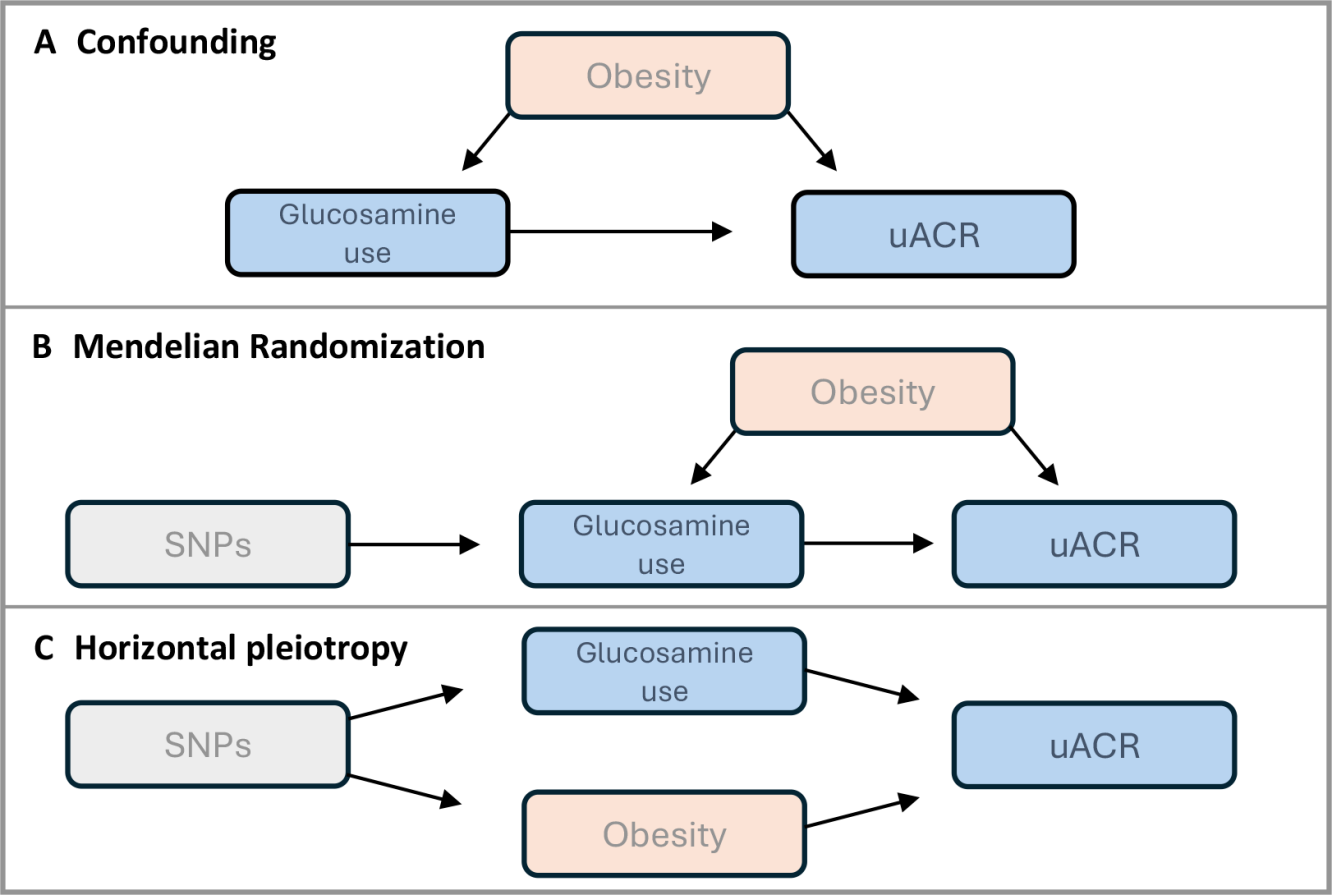
Examples of directed acyclic graphs of glucosamine use and uACR. (A) Confounding, (B) Mendelian randomisation, (C) Horizontal pleiotropy. SNPs, single-nucleotide polymorphisms; uACR, urine albumin creatinine ratio.

Glucosamine is a low-cost and well-tolerated medication. Our study adds to the body of evidence linking glucosamine with positive health outcomes and identifies a possible underlying biological mechanism. Glucosamine has never been examined in relation to vascular outcomes in a randomised control trial; this is most likely because of the length of follow-up that would be required to observe these outcomes. In the presence of the positive observational evidence but practical limitations of conducting a randomised control trial, MR analyses would be perfectly placed to examine whether there is a causal link between glucosamine and vascular outcomes, were appropriate summary genetic data available. The key benefit of MR analyses is that they reduce the bias from confounding factors. For example, obesity could be confounding the observational analysis as obesity can lead to joint problems, for which people might take glucosamine, and obesity is associated with albuminuria (figure 3A). Even after adjusting for obesity measures in our observational analysis, residual confounding may still be at play. The MR analyses aim to reduce this bias (figure 3B).

However, as there are currently no GWAS studies of circulating glucosamine levels, SNPs that accurately proxy for glucosamine levels cannot be identified. Instead, our and other published studies have used genetic proxies for glucosamine use (not circulating levels) in MR analyses. Glucosamine use is a distal and non-ubiquitous exposure and thus MR analyses using these genetic proxies are likely to suffer from horizontal pleiotropy (figure 3C), as many biological mechanisms may be disrupted on the pathway between germline genetic variants and future supplement use. Indeed, the 5 SNPs used as genetic instruments for glucosamine use do not have a known biological relationship with glucosamine synthesis or metabolism pathways. Demonstrating these issues, our phenome-wide association analysis identified 211 exposures associated with genetically proxied glucosamine use, including possible horizontally pleiotropic pathways such as alcohol consumption and adiposity. It is plausible that using SNPs that proxy glucosamine levels may also suffer from horizontal pleiotropy; however, we would expect this to be much reduced as glucosamine level is a more specific trait than glucosamine use. Therefore, we urge that the findings of such analyses must be interpreted accordingly.

In our MR analysis, we found little evidence for a causal effect of genetic predisposition to glucosamine use on albuminuria. It is crucial that we, alongside other glucosamine studies which have used MR in this way, are fully transparent with the interpretation of these findings and do not draw erroneous conclusions about the causal effect of glucosamine levels on the outcome of interest. Given the breadth of observational glucosamine studies reporting associations, we believe that a GWAS of circulating glucosamine levels is warranted and would allow MR to be performed in a variety of disease settings. Indeed, a validated method to detect endogenous glucosamine exists and has demonstrated large variation between individuals but minimal variation within individuals.^41^

The strengths of our study include the large sample size, use of clinically relevant uACR categories and consistent findings across all subgroup and sensitivity analyses. As explained throughout, there are several limitations of using MR with currently available GWAS summary data. In addition, there are several further limitations to consider. First, our use of UKBB data means there may be participation bias underlying our analyses, given UKBB participants have been shown previously to be less likely to have obesity, smoke or drink alcohol compared with the UK general population.^42^ Therefore, our findings may not be generalisable to the wider population. Second, glucosamine use was self-reported and we do not have details of the patterns of use. For example, we do not know the length of time that people were on glucosamine or if ‘non-users’ had ever taken the medication. Third, our observational findings could be the result of unmeasured or residual confounding. For example, people who take glucosamine may be more engaged with their health, have certain healthy behaviours or take other supplementary treatments which have had a positive effect on albuminuria. There are approaches for assessing residual confounding, such as reporting E-values; however, these approaches have their own limitations. For example, there is little consensus across the literature as to what indicates a high or low enough E-value to draw a meaningful conclusion about residual confounding.^43 44^ Fourth, in the MR analysis, the datasets used for the albuminuria GWAS (CKDGen) and glucosamine use GWAS (UKBB) could contain overlapping participants as the UK was a recruiting country to CKDGen. However, it is likely that the overlap, if present, is minimal. Fifth, the MR analyses were limited to people of European ancestry and so this limits the generalisability of these results. Sixth, the instrumental variable in the MR contained only five SNPs and so may have been underpowered; although relaxing the p value threshold for SNP selection could have increased power, it would also have likely introduced weak instrument bias.^45^ Finally, the independence and exclusion restriction MR assumptions are ultimately unverifiable and therefore cannot be proven.

In conclusion, we found an association between glucosamine use and lower albuminuria levels. Although we cannot establish if this is a causal relationship, it is plausible that glucosamine could protect the vascular endothelium within the glomerular filtration barrier via its action on the glycocalyx. We believe this hypothesis warrants further investigation. A randomised controlled trial would be the optimal approach to establish a causal role of glucosamine in reduction of albuminuria, but in view of the challenges in conducting such a trial, MR offers a promising next step. We highlight the need for a GWAS of endogenous glucosamine levels to support such analyses.

## Supplementary material

10.1136/bmjopen-2024-096344 online supplemental file 1

## Data Availability

Data may be obtained from a third party and are not publicly available.
