# Supplementary material for: Association between glucosamine use and albuminuria in the UK: a cohort and Mendelian randomisation study
Source: BMJ Open. 2025 Nov 21;15(11):e096344. doi: 10.1136/bmjopen-2024-096344 (PMC12658525; doi:10.1136/bmjopen-2024-096344)
Supplement: online supplemental file 1 [file bmjopen-15-11-s001.docx]

***Supplementary Tables***

| **Supplementary Table 1. Information from the UK Biobank genome wide association studies used to identify genetic instruments** | | | |
| --- | --- | --- | --- |
| **Outcome definition** | **Number of people** | **Number of SNPs** | **SNPs selected as instrumental variables** |
| Do you take any of the following?  Glucosamine selected from a list of supplements | 461,384 | 9,851,867 | rs11915360  rs1466978  rs34084719  rs2565185  rs735465 |
| **Abbreviations:** SNP single nucleotide polymorphism. | | | |

| **Supplementary Table 2. Information on genetic instruments** | | | | | | | | | | | | |
| --- | --- | --- | --- | --- | --- | --- | --- | --- | --- | --- | --- | --- |
| **SNP** | **Effect allele** | **Other allele** | **Glucosamine use** | | | | | | **uACR** | | | |
|  |  |  | **Effect allele frequency** | **Beta** | **Standard error** | **P value** | **F statistic** | **R^2^** | **Effect allele frequency** | **Beta** | **Standard error** | **P value** |
| rs11915360 | T | C | 0.57 | -0.005 | 0.001 | 8.00 X 10^-9^ | 33.27 | 7.21 X 10^-5^ | 0.56 | 0.017 | 0.020 | 0.40 |
| rs1466978 | C | T | 0.76 | 0.006 | 0.001 | 1.30 X 10^-9^ | 36.88 | 7.99 X 10^-5^ | 0.77 | 0.048 | 0.025 | 0.06 |
| rs2565185 | C | T | 0.28 | -0.005 | 0.001 | 1.70 X 10^-8^ | 31.77 | 6.89 X 10^-5^ | 0.29 | -0.002 | 0.025 | 0.07 |
| rs34084719 | C | T | 0.21 | 0.006 | 0.001 | 9.70 X 10^-9^ | 32.90 | 7.13 X 10^-5^ | 0.18 | 0.006 | 0.028 | 0.83 |
| rs735465 | G | C | 0.76 | 0.005 | 0.001 | 1.30 X 10^-8^ | 32.33 | 7.01 X 10^-5^ | 0.75 | -0.006 | 0.027 | 0.84 |
| **Abbreviations:** SNP single nucleotide polymorphism, uACR urinary albumin creatinine ratio. | | | | | | | | | | | | |

| **Supplementary Table 3. Sensitivity analyses results – association between glucosamine use and uACR analysed as a continuous variable.** | | | | |
| --- | --- | --- | --- | --- |
| **Model** | **Beta** | **95% CIs** | **Standard error** | **P value** |
| uACR as a continuous variable  Univariable linear regression  (uACR log transformed) | -0.01 | -0.01 - -0.01 | 0.002 | 1.41 x 10^-5^ |
| uACR as a continuous variable  Univariable linear regression  (uACR rank inverse normalisation) | -0.06 | -0.07 - -0.06 | 0.003 | <2.2 x 10^-16^ |
| **Abbreviations:** CIs confidence intervals, uACR urinary albumin creatinine ratio. | | | | |

| **Supplementary Table 5. Subgroup analyses results - association between glucosamine use and uACR adjusted for age, sex, body mass index and waist to hip ratio.** | | | |
| --- | --- | --- | --- |
| **Model** | **OR** | **95% CIs** | **P value** |
| Diabetic participants only (n=22,043) | 0.87 | 0.81-0.93 | 0.00017 |
| Non-diabetic participants only (n=414,149) | 0.81 | 0.80-0.83 | <2.2 x 10^-16^ |
| Participants with eGFR < 60 only (n=9,619) | 0.78 | 0.71-0.87 | 3.73 x 10^-6^ |
| Participants with eGFR ≥ 60 only (n=405,913) | 0.81 | 0.80-0.82 | <2.2 x 10^-16^ |
| **Abbreviations:** CIs confidence intervals, eGFR estimated glomerular filtration rate, OR odds ratio, uACR urinary albumin creatinine ratio. | | | |

| **Supplementary Table 4. Sensitivity analyses results – association between glucosamine use and uACR adjusted for age, sex, body mass index and waist to hip ratio, as well as smoking and blood pressure.** | | | |
| --- | --- | --- | --- |
| **Model** | **OR** | **95% CIs** | **P value** |
| Multivariable ordinal logistic regression | 0.81 | 0.80-0.83 | <2.2 x 10^-16^ |
| **Abbreviations:** CIs confidence intervals, OR odds ratio, uACR urinary albumin creatinine ratio. | | | |

| **Supplementary Table 6. OpenGWAS PheWAS results for SNP rs11915360 for traits which met the predefined *P*-value threshold of < 9.99 x 10^-7^.**Columns are as reported by OpenGWAS, where: ID = OpenGWAS GWAS ID, Trait = name of trait (may be Ensembl ID for gene expression), Position = genomic position, P = *P*-value of SNP-trait association, SE = standard error, N = sample size (where given on OpenGWAS), Beta = beta of SNP-trait association, CHR = chromosome, EA = effect alelle, NEA = other allele, EAF = effect allele frequency. | | | | | | | | | | |
| --- | --- | --- | --- | --- | --- | --- | --- | --- | --- | --- |
| **ID** | **Trait** | **Position** | **P** | **SE** | **N** | **Beta** | **CHR** | **EA** | **NEA** | **EAF** |
| eqtl-a-ENSG00000163320 | ENSG00000163320 | 88643136 | 5.2076E-09 | 0.0119382 | 31563 | 0.0697239 | 3 | T | C | 0.545285 |
| ukb-b-14238 | Mineral and other dietary supplements: None of the above | 88643136 | 5.3001E-09 | 0.00103766 | 461384 | 0.00605804 | 3 | T | C | 0.570853 |
| ukb-b-11535 | Mineral and other dietary supplements: Glucosamine | 88643136 | 8E-09 | 0.00082916 | 461384 | -0.0047827 | 3 | T | C | 0.570853 |
| ukb-b-11075 | Mineral and other dietary supplements: Fish oil (including cod liver oil) | 88643136 | 1.2E-08 | 0.00097808 | 461384 | -0.0055809 | 3 | T | C | 0.570853 |
| ukb-a-494 | Mineral and other dietary supplements: Glucosamine | 88643136 | 1.574E-08 | 0.0009774 | 336314 | -0.0055257 | 3 | T | C | 0.573789 |
| ubm-b-308 | ThalamNuclei_lh_volume_Pc | 88643136 | 1.5996E-08 | 0.0080672 |  | 0.045596 | 3 | T | C | 0.57474 |
| ubm-b-324 | ThalamNuclei_rh_volume_VA | 88643136 | 7.7607E-08 | 0.0080676 |  | 0.043354 | 3 | T | C | 0.57474 |
| ubm-b-1653 | IDP_dMRI_TBSS_L1_Body_of_corpus_callosum | 88643136 | 9.23E-08 | 0.0081423 |  | -0.043501 | 3 | T | C | 0.57474 |
| ubm-b-300 | ThalamNuclei_lh_volume_CeM | 88643136 | 2.596E-07 | 0.0080679 |  | 0.041564 | 3 | T | C | 0.57474 |
| ubm-b-1317 | aparc-a2009s_rh_thickness_S-pericallosal | 88643136 | 2.6044E-07 | 0.0080679 |  | 0.041559 | 3 | T | C | 0.57474 |
| ubm-b-298 | ThalamNuclei_lh_volume_VAmc | 88643136 | 3.2659E-07 | 0.008068 |  | 0.041215 | 3 | T | C | 0.57474 |
| ubm-b-318 | ThalamNuclei_rh_volume_VLa | 88643136 | 7.9983E-07 | 0.0080682 |  | 0.039828 | 3 | T | C | 0.57474 |
| ubm-b-326 | ThalamNuclei_rh_volume_CeM | 88643136 | 8.4159E-07 | 0.0080682 |  | 0.039747 | 3 | T | C | 0.57474 |

| **Supplementary Table 7. OpenGWAS PheWAS results for SNP rs1466978 for traits which met the predefined *P*-value threshold of < 9.99 x 10^-7^.** Columns are as reported by OpenGWAS, where: ID = OpenGWAS GWAS ID, Trait = name of trait (may be Ensembl ID for gene expression), Position = genomic position, P = *P*-value of SNP-trait association, SE = standard error, N = sample size (where given on OpenGWAS), Beta = beta of SNP-trait association, CHR = chromosome, EA = effect alelle, NEA = other allele, EAF = effect allele frequency (where available). | | | | | | | | | | |
| --- | --- | --- | --- | --- | --- | --- | --- | --- | --- | --- |
| **ID** | **Trait** | **Position** | **P** | **SE** | **N** | **Beta** | **CHR** | **EA** | **NEA** | **EAF** |
| eqtl-a-ENSG00000109133 | ENSG00000109133 | 41983544 | 7.6173E-16 | 0.0141068 | 30867 | -0.113705 | 4 | C | T | 0.769833 |
| ukb-b-14238 | Mineral and other dietary supplements: None of the above | 41983544 | 4.9E-11 | 0.00120399 | 461384 | -0.0079156 | 4 | C | T | 0.761361 |
| ukb-b-11535 | Mineral and other dietary supplements: Glucosamine | 41983544 | 1.3E-09 | 0.00096206 | 461384 | 0.00584216 | 4 | C | T | 0.761361 |
| eqtl-a-ENSG00000188848 | ENSG00000188848 | 41983544 | 3.0875E-08 | 0.0141239 | 25692 | 0.0781954 | 4 | C | T | 0.769833 |
| ukb-b-11075 | Mineral and other dietary supplements: Fish oil (including cod liver oil) | 41983544 | 9.2001E-08 | 0.00113486 | 461384 | 0.00606272 | 4 | C | T | 0.761361 |
| ukb-b-17381 | Vitamin and mineral supplements: None of the above | 41983544 | 1.1E-07 | 0.00113022 | 460351 | -0.0059942 | 4 | C | T | 0.761309 |
| ukb-b-7376 | Impedance of leg (right) | 41983544 | 2.2E-07 | 0.00211014 | 454863 | -0.0109306 | 4 | C | T | 0.761265 |
| ukb-b-14068 | Impedance of leg (left) | 41983544 | 2.9E-07 | 0.00212824 | 454857 | -0.0109243 | 4 | C | T | 0.761262 |
| ebi-a-GCST90096909 | Dried fruit consumption | 41983544 | 6.3062E-07 | 0.00144852 | 409125 | 0.00721594 | 4 | C | T |  |
| ukb-b-16878 | Alcohol usually taken with meals | 41983544 | 8.0999E-07 | 0.00154269 | 235645 | 0.00760908 | 4 | C | T | 0.761637 |

| **Supplementary Table 8. OpenGWAS PheWAS results for SNP rs34084719 for traits which met the predefined *P*-value threshold of < 9.99 x 10^-7^.** Columns are as reported by OpenGWAS, where: ID = OpenGWAS GWAS ID, Trait = name of trait (may be Ensembl ID for gene expression), Position = genomic position, P = *P*-value of SNP-trait association, SE = standard error, N = sample size (where given on OpenGWAS), Beta = beta of SNP-trait association, CHR = chromosome, EA = effect alelle, NEA = other allele, EAF = effect allele frequency (where available). Note that a *P*-value of 0 reflects a rounding of *P*-value < 1E-300. | | | | | | | | | | |
| --- | --- | --- | --- | --- | --- | --- | --- | --- | --- | --- |
| **ID** | **Trait** | **Position** | **P** | **SE** | **N** | **Beta** | **CHR** | **EA** | **NEA** | **EAF** |
| eqtl-a-ENSG00000164597 | ENSG00000164597 | 107095227 | 0 | 0.0143043 | 31355 | -0.555658 | 7 | C | T | 0.194063 |
| eqtl-a-ENSG00000241764 | ENSG00000241764 | 107095227 | 2.2331E-35 | 0.0149681 | 22495 | 0.185794 | 7 | C | T | 0.194063 |
| ebi-a-GCST90013991 | Alkaline phosphatase levels (UKB data field 30610) | 107095227 | 1.7503E-22 | 0.00242775 | 389883 | -0.0236835 | 7 | C | T |  |
| ebi-a-GCST90018942 | Serum alkaline phosphatase levels | 107095227 | 3.6728E-22 | 0.0026 | 344292 | -0.0255 | 7 | C | T | 0.180468 |
| ukb-d-30610_irnt | Alkaline phosphatase | 107095227 | 1.4846E-15 | 0.0028583 | 344292 | -0.022805 | 7 | C | T | 0.21098 |
| ieu-b-107 | apolipoprotein A-I | 107095227 | 6.5993E-15 | 0.0024151 |  | 0.0188196 | 7 | C | T | 0.210172 |
| ukb-d-30610_raw | Alkaline phosphatase | 107095227 | 1.5535E-13 | 0.076556 | 344292 | -0.5652 | 7 | C | T | 0.21098 |
| ebi-a-GCST90013993 | Apolipoprotein A levels (UKB data field 30630) | 107095227 | 3.1586E-12 | 0.00248453 | 355859 | 0.0173184 | 7 | C | T |  |
| ieu-b-109 | HDL cholesterol | 107095227 | 9.3994E-12 | 0.00234528 |  | 0.0159833 | 7 | C | T | 0.210198 |
| ukb-b-4616 | Nap during day | 107095227 | 1.3E-09 | 0.00150513 | 462400 | 0.00913217 | 7 | C | T | 0.210244 |
| ebi-a-GCST90018722 | Serum alkaline phosphatase levels | 107095227 | 2.3E-09 | 0.00671634 | 118886 | -0.0401087 | 7 | C | T | 0.092105 |
| ukb-d-30630_irnt | Apoliprotein A | 107095227 | 2.3145E-09 | 0.0028459 | 313387 | 0.017002 | 7 | C | T | 0.2108 |
| ebi-a-GCST90014007 | High density lipoprotein cholesterol levels (UKB data field 30760) | 107095227 | 2.8315E-09 | 0.00239609 | 357810 | 0.0142353 | 7 | C | T |  |
| ukb-a-12 | Nap during day | 107095227 | 2.9788E-09 | 0.00176784 | 337074 | 0.0104885 | 7 | C | T | 0.211553 |
| ukb-b-11535 | Mineral and other dietary supplements: Glucosamine | 107095227 | 9.7E-09 | 0.00100813 | 461384 | 0.00578287 | 7 | C | T | 0.210243 |
| eqtl-a-ENSG00000105856 | ENSG00000105856 | 107095227 | 1.2674E-08 | 0.0150323 | 31355 | 0.0855398 | 7 | C | T | 0.194063 |
| ebi-a-GCST011494 | Daytime nap | 107095227 | 1.4E-08 | 0.00149165 | 452633 | 0.00851927 | 7 | C | T | 0.210188 |
| ukb-d-30630_raw | Apoliprotein A | 107095227 | 1.5312E-08 | 0.00077186 | 313387 | 0.0043673 | 7 | C | T | 0.2108 |
| ukb-b-14238 | Mineral and other dietary supplements: None of the above | 107095227 | 2.1E-08 | 0.0012617 | 461384 | -0.0070712 | 7 | C | T | 0.210243 |
| ebi-a-GCST90018956 | HDL cholesterol | 107095227 | 5.6571E-08 | 0.0026 | 315133 | 0.0144 | 7 | C | T | 0.187893 |
| bbj-a-5 | Alkaline phosphatase | 107095227 | 8.844E-08 | 0.007567 |  | -0.04047 | 7 | C | T | 0.0913 |
| ukb-b-4424 | Sleep duration | 107095227 | 2.4E-07 | 0.00196608 | 460099 | 0.0101575 | 7 | C | T | 0.210267 |
| ebi-a-GCST90002412 | Low density lipoprotein cholesterol levels | 107095227 | 4.9E-07 | 0.0023524 | 431167 | -0.0118375 | 7 | C | T | 0.210428 |
| ukb-d-30760_irnt | HDL cholesterol | 107095227 | 5.964E-07 | 0.0027979 | 315133 | 0.013968 | 7 | C | T | 0.21083 |
| ukb-b-2122 | Ankle spacing width (left) | 107095227 | 0.00000064 | 0.00379657 | 146226 | -0.0189039 | 7 | C | T | 0.210256 |

| **Supplementary Table 9. OpenGWAS PheWAS results for SNP rs2565185 for traits which met the predefined *P*-value threshold of < 9.99 x 10^-7^.** Columns are as reported by OpenGWAS, where: ID = OpenGWAS GWAS ID, Trait = name of trait (may be Ensembl ID for gene expression), Position = genomic position, P = *P*-value of SNP-trait association, SE = standard error, N = sample size (where given on OpenGWAS), Beta = beta of SNP-trait association, CHR = chromosome, EA = effect alelle, NEA = other allele, EAF = effect allele frequency (where available). | | | | | | | | | | |
| --- | --- | --- | --- | --- | --- | --- | --- | --- | --- | --- |
| **ID** | **Trait** | **Position** | **P** | **SE** | **N** | **Beta** | **CHR** | **EA** | **NEA** | **EAF** |
| ebi-a-GCST90028996 | Mean platelet volume | 65392056 | 6.295E-307 | 0.00195577 | 694866 | 0.0701437 | 10 | C | T | 0.280534 |
| ebi-a-GCST90013981 | Mean platelet thrombocyte volume (UKB data field 30100) | 65392056 | 1.377E-250 | 0.00211861 | 396616 | 0.0716315 | 10 | C | T |  |
| ebi-a-GCST90002346 | Mean platelet volume | 65392056 | 1.879E-250 | 0.002139 | 460935 | 0.072312 | 10 | C | T | 0.279095 |
| ebi-a-GCST90002395 | Mean platelet volume | 65392056 | 2.897E-224 | 0.00228314 | 408112 | 0.0729898 | 10 | C | T | 0.279734 |
| ebi-a-GCST90012110 | Sex hormone-binding globulin levels adjusted for BMI | 65392056 | 9.908E-206 | 0.00092734 | 368929 | -0.0272279 | 10 | C | T | 0.280998 |
| ebi-a-GCST90012111 | Sex hormone-binding globulin levels | 65392056 | 1.901E-189 | 0.00103369 | 370125 | -0.0291132 | 10 | C | T | 0.280931 |
| ukb-d-30100_irnt | Mean platelet (thrombocyte) volume | 65392056 | 4.467E-162 | 0.00265552 | 350470 | 0.0720797 | 10 | C | T | 0.281351 |
| ebi-a-GCST90012108 | Sex hormone-binding globulin levels adjusted for BMI | 65392056 | 1.3E-148 | 0.00127195 | 180094 | -0.0317679 | 10 | C | T | 0.281019 |
| ebi-a-GCST90014011 | Sex hormone binding globulin levels (UKB data field 30830) | 65392056 | 2.78E-144 | 0.0022077 | 354620 | -0.0564655 | 10 | C | T |  |
| ebi-a-GCST90012109 | Sex hormone-binding globulin levels | 65392056 | 5.395E-139 | 0.00135868 | 180726 | -0.0327734 | 10 | C | T | 0.280958 |
| ieu-b-4871 | Sex hormone binding globulin (SHBG) | 65392056 | 2.099E-111 | 0.0035158 |  | -0.0788487 | 10 | C | T | 0.280316 |
| ebi-a-GCST90013991 | Alkaline phosphatase levels (UKB data field 30610) | 65392056 | 1.469E-108 | 0.00220825 | 389883 | -0.0488787 | 10 | C | T |  |
| ukb-d-30830_irnt | SHBG | 65392056 | 6.138E-103 | 0.002523 | 312215 | -0.054374 | 10 | C | T | 0.28116 |
| ebi-a-GCST90028999 | Platelet count | 65392056 | 3.8E-100 | 0.00208983 | 600968 | -0.0422092 | 10 | C | T | 0.280554 |
| ebi-a-GCST90002357 | Platelet count | 65392056 | 9.6806E-98 | 0.002048 | 542827 | -0.042974 | 10 | C | T | 0.276514 |
| eqtl-a-ENSG00000148572 | ENSG00000148572 | 65392056 | 1.2232E-81 | 0.0136507 | 14263 | -0.261242 | 10 | C | T | 0.245622 |
| ebi-a-GCST90002402 | Platelet count | 65392056 | 6.9008E-78 | 0.00235167 | 408112 | -0.043934 | 10 | C | T | 0.279733 |
| ebi-a-GCST90013980 | Platelet count (UKB data field 30080) | 65392056 | 2.6829E-73 | 0.00219715 | 396621 | -0.0397892 | 10 | C | T |  |
| ukb-d-30610_irnt | Alkaline phosphatase | 65392056 | 8.6736E-72 | 0.0025908 | 344292 | -0.046431 | 10 | C | T | 0.28128 |
| ebi-a-GCST90018942 | Serum alkaline phosphatase levels | 65392056 | 1.6259E-69 | 0.0026 | 344292 | -0.0449 | 10 | C | T | 0.213772 |
| ukb-d-30830_raw | SHBG | 65392056 | 3.5253E-69 | 0.070689 | 312215 | -1.243 | 10 | C | T | 0.28116 |
| ebi-a-GCST004599 | Mean platelet volume | 65392056 | 1.8789E-68 | 0.0040297 | 164454 | 0.0704575 | 10 | C | T | 0.2785 |
| ebi-a-GCST90012106 | Sex hormone-binding globulin levels adjusted for BMI | 65392056 | 1.5999E-65 | 0.00137348 | 188908 | -0.0217219 | 10 | C | T | 0.280984 |
| ebi-a-GCST90012113 | Total testosterone levels | 65392056 | 2.8003E-60 | 0.00336604 | 194453 | -0.0533023 | 10 | C | T | 0.28131 |
| ebi-a-GCST90012107 | Sex hormone-binding globulin levels | 65392056 | 7.5007E-60 | 0.00157937 | 189473 | -0.0243388 | 10 | C | T | 0.280911 |
| eqtl-a-ENSG00000165476 | ENSG00000165476 | 65392056 | 1.205E-59 | 0.0136985 | 26494 | -0.223116 | 10 | C | T | 0.245622 |
| ukb-d-30080_irnt | Platelet count | 65392056 | 1.845E-55 | 0.00257225 | 350474 | -0.0403589 | 10 | C | T | 0.281349 |
| ieu-b-4865 | Total Testosterone | 65392056 | 1.9999E-55 | 0.00344186 |  | -0.053973 | 10 | C | T | 0.280539 |
| ebi-a-GCST90018969 | Platelet count | 65392056 | 2.7328E-53 | 0.0025 | 350474 | -0.0387 | 10 | C | T | 0.202991 |
| ieu-b-4870 | Sex hormone binding globulin (SHBG) | 65392056 | 1.3999E-49 | 0.00329441 |  | -0.0487619 | 10 | C | T | 0.280126 |
| ukb-d-30610_raw | Alkaline phosphatase | 65392056 | 3.2659E-49 | 0.069403 | 344292 | -1.0236 | 10 | C | T | 0.28128 |
| ukb-d-30850_raw | Testosterone | 65392056 | 1.0503E-41 | 0.0075312 | 312102 | -0.10191 | 10 | C | T | 0.28117 |
| ebi-a-GCST90012114 | Total testosterone levels | 65392056 | 9.3004E-37 | 0.00143063 | 425097 | -0.0177858 | 10 | C | T | 0.281032 |
| ebi-a-GCST90014013 | Testosterone levels (UKB data field 30850) | 65392056 | 3.6058E-31 | 0.00161372 | 353805 | -0.0187376 | 10 | C | T |  |
| ebi-a-GCST90026372 | Mitochondrial DNA copy number | 65392056 | 8.0076E-31 | 0.0024 | 383476 | 0.0272 | 10 | C | T |  |
| ebi-a-GCST90026371 | Mitochondrial DNA copy number | 65392056 | 1.7231E-30 | 0.0023 | 395718 | 0.0267 | 10 | C | T |  |
| ukb-d-30850_irnt | Testosterone | 65392056 | 3.2122E-30 | 0.0016996 | 312102 | -0.019417 | 10 | C | T | 0.28117 |
| ebi-a-GCST90013872 | Total bilirubin levels (UKB data field 30840) | 65392056 | 4.4432E-25 | 0.00198777 | 388303 | 0.0205621 | 10 | C | T |  |
| ebi-a-GCST90014012 | Total bilirubin levels (UKB data field 30840) | 65392056 | 6.8691E-25 | 0.00200148 | 388303 | 0.0206202 | 10 | C | T |  |
| ebi-a-GCST004603 | Platelet count | 65392056 | 1.8399E-24 | 0.00406464 | 166066 | -0.0414888 | 10 | C | T | 0.2788 |
| ebi-a-GCST90029003 | White blood cell count | 65392056 | 7.8001E-20 | 0.00219714 | 503190 | 0.0192799 | 10 | C | T | 0.280552 |
| ukb-d-30840_raw | Total bilirubin | 65392056 | 6.8375E-19 | 0.011543 | 342829 | 0.10248 | 10 | C | T | 0.28135 |
| ukb-d-30840_irnt | Total bilirubin | 65392056 | 1.7787E-18 | 0.0025608 | 342829 | 0.022461 | 10 | C | T | 0.28135 |
| ebi-a-GCST90029008 | Height | 65392056 | 6.7999E-18 | 0.00175068 | 673878 | -0.0147849 | 10 | C | T | 0.280529 |
| ebi-a-GCST90018973 | Total bilirubin levels | 65392056 | 9.2003E-18 | 0.0025 | 342829 | 0.0216 | 10 | C | T | 0.211366 |
| ukb-b-10787 | Standing height | 65392056 | 4.3003E-16 | 0.00145791 | 461950 | -0.0118534 | 10 | C | T | 0.280416 |
| ebi-a-GCST90013976 | White blood cell leukocyte count (UKB data field 30000) | 65392056 | 1.7677E-15 | 0.00237557 | 396621 | 0.0189016 | 10 | C | T |  |
| ebi-a-GCST90029000 | Platelet distribution width | 65392056 | 3.1996E-15 | 0.00214622 | 572201 | 0.0167218 | 10 | C | T | 0.280564 |
| ieu-b-30 | white blood cell count | 65392056 | 6.6206E-14 | 0.002055 | 561301 | 0.015418 | 10 | C | T | 0.277424 |
| ebi-a-GCST90002374 | White blood cell count | 65392056 | 6.6206E-14 | 0.002055 | 562243 | 0.015418 | 10 | C | T | 0.277424 |
| ieu-b-111 | triglycerides | 65392056 | 7.1995E-14 | 0.00221409 |  | 0.016571 | 10 | C | T | 0.28031 |
| ebi-a-GCST90013984 | Neutrophill count (UKB data field 30140) | 65392056 | 2.1208E-13 | 0.00238693 | 395949 | 0.0175223 | 10 | C | T |  |
| ebi-a-GCST90013997 | Direct bilirubin levels (UKB data field 30660) | 65392056 | 2.8119E-13 | 0.00231466 | 332739 | 0.0169042 | 10 | C | T |  |
| ebi-a-GCST90018975 | Triglycerides | 65392056 | 2.8602E-13 | 0.0025 | 343992 | 0.0186 | 10 | C | T | 0.216794 |
| ebi-a-GCST90028994 | White blood cell count (lymphocyte) | 65392056 | 3.2999E-13 | 0.00219546 | 509277 | 0.0151682 | 10 | C | T | 0.280575 |
| met-d-XS_VLDL_CE_pct | Cholesteryl esters to total lipids ratio in very small VLDL | 65392056 | 1.2001E-12 | 0.00435036 |  | -0.0291023 | 10 | C | T | 0.279366 |
| ukb-d-30870_irnt | Triglycerides | 65392056 | 1.6237E-12 | 0.0025838 | 343992 | 0.018251 | 10 | C | T | 0.28127 |
| ieu-a-1006 | Mean platelet volume | 65392056 | 2.0502E-12 | 0.0013 | 16239 | 0.0093 | 10 | C | T |  |
| ukb-d-30660_raw | Direct bilirubin | 65392056 | 7.5823E-12 | 0.0024199 | 292933 | 0.016568 | 10 | C | T | 0.28203 |
| ebi-a-GCST90018978 | White blood cell count | 65392056 | 8.0242E-12 | 0.0026 | 350470 | 0.0177 | 10 | C | T | 0.200915 |
| met-d-XS_VLDL_C_pct | Cholesterol to total lipids ratio in very small VLDL | 65392056 | 8.1003E-12 | 0.00437327 |  | -0.0279844 | 10 | C | T | 0.279366 |
| ukb-d-30000_irnt | White blood cell (leukocyte) count | 65392056 | 9.4886E-12 | 0.00265024 | 350470 | 0.0180595 | 10 | C | T | 0.281346 |
| ebi-a-GCST90014014 | Triglyceride levels (UKB data field 30870) | 65392056 | 1.2173E-11 | 0.00227935 | 389562 | 0.0154498 | 10 | C | T |  |
| met-d-L_LDL_FC_pct | Free cholesterol to total lipids ratio in large LDL | 65392056 | 1.3999E-11 | 0.00439868 |  | -0.0276621 | 10 | C | T | 0.279366 |
| ebi-a-GCST90012067 | Proheparin-binding EGF-like growth factor levels | 65392056 | 2.8067E-11 | 0.0121 | 21758 | -0.0804 | 10 | C | T | 0.2564 |
| ieu-b-34 | neutrophil cell count | 65392056 | 3.8504E-11 | 0.002143 | 518347 | 0.014173 | 10 | C | T | 0.278869 |
| ebi-a-GCST90002351 | Neutrophil count | 65392056 | 3.8504E-11 | 0.002143 | 519288 | 0.014173 | 10 | C | T | 0.278869 |
| met-d-Unsaturation | Degree of unsaturation | 65392056 | 4.1995E-11 | 0.00438821 |  | -0.0272374 | 10 | C | T | 0.279371 |
| ebi-a-GCST90093031 | Cholesteryl esters to total lipids ratio in very small VLDL | 65392056 | 6.0996E-11 | 0.00432357 | 115082 | -0.0282787 | 10 | C | T | 0.27936 |
| ukb-d-30870_raw | Triglycerides | 65392056 | 1.6046E-10 | 0.0026696 | 343992 | 0.017073 | 10 | C | T | 0.28127 |
| ebi-a-GCST90013999 | Calcium levels (UKB data field 30680) | 65392056 | 1.6129E-10 | 0.002521 | 357831 | 0.01612 | 10 | C | T |  |
| ukb-d-30110_irnt | Platelet distribution width | 65392056 | 1.6911E-10 | 0.002631 | 350470 | 0.0168048 | 10 | C | T | 0.281351 |
| met-d-XS_VLDL_TG_pct | Triglycerides to total lipids ratio in very small VLDL | 65392056 | 1.9E-10 | 0.00438391 |  | 0.0264205 | 10 | C | T | 0.279366 |
| ebi-a-GCST90013982 | Lymphocyte count (UKB data field 30120) | 65392056 | 2.6628E-10 | 0.00236195 | 395949 | 0.014921 | 10 | C | T |  |
| ebi-a-GCST90018968 | Neutrophil count | 65392056 | 2.852E-10 | 0.0026 | 349856 | 0.0165 | 10 | C | T | 0.231067 |
| ukb-d-30140_irnt | Neutrophill count | 65392056 | 3.3025E-10 | 0.00264652 | 349856 | 0.0166309 | 10 | C | T | 0.281357 |
| ebi-a-GCST90092994 | Degree of unsaturation | 65392056 | 3.6E-10 | 0.00437314 | 115006 | -0.0274167 | 10 | C | T | 0.279367 |
| ebi-a-GCST90093029 | Cholesterol to total lipids ratio in very small VLDL | 65392056 | 4E-10 | 0.00435236 | 115082 | -0.0272131 | 10 | C | T | 0.27936 |
| ebi-a-GCST90002407 | White blood cell count | 65392056 | 4.1E-10 | 0.00236374 | 408112 | 0.0147771 | 10 | C | T | 0.280814 |
| ebi-a-GCST90012102 | Bioavailable testosterone levels | 65392056 | 6.5E-10 | 0.00282398 | 188507 | 0.0167615 | 10 | C | T | 0.280846 |
| ebi-a-GCST90092861 | Free cholesterol to total lipids ratio in large LDL | 65392056 | 6.9E-10 | 0.00437685 | 115082 | -0.0270013 | 10 | C | T | 0.27936 |
| ukb-d-30660_irnt | Direct bilirubin | 65392056 | 8.0149E-10 | 0.0027822 | 292933 | 0.017096 | 10 | C | T | 0.28203 |
| ebi-a-GCST90092932 | Ratio of omega-3 fatty acids to total fatty acids | 65392056 | 9.7E-10 | 0.00442623 | 115006 | -0.0270608 | 10 | C | T | 0.279367 |
| ukb-b-7992 | Diastolic blood pressure, automated reading | 65392056 | 1.2E-09 | 0.00230584 | 436424 | 0.0140168 | 10 | C | T | 0.279656 |
| met-d-Omega_3_pct | Ratio of omega-3 fatty acids to total fatty acids | 65392056 | 1.2E-09 | 0.00453057 |  | -0.0265325 | 10 | C | T | 0.279371 |
| met-d-DHA_pct | Ratio of docosahexaenoic acid to total fatty acids | 65392056 | 1.8E-09 | 0.00448736 |  | -0.0262176 | 10 | C | T | 0.279371 |
| ebi-a-GCST90014006 | Glycated haemoglobin HbA1c levels (UKB data field 30750) | 65392056 | 2.5117E-09 | 0.00226884 | 389889 | 0.0135239 | 10 | C | T |  |
| ebi-a-GCST004610 | White blood cell count | 65392056 | 2.654E-09 | 0.00399182 | 172435 | 0.0237579 | 10 | C | T | 0.2795 |
| ebi-a-GCST90000025 | Appendicular lean mass | 65392056 | 2.851E-09 | 0.0021 | 450243 | -0.0125 | 10 | C | T | 0.2806 |
| met-d-IDL_C_pct | Cholesterol to total lipids ratio in IDL | 65392056 | 3.5E-09 | 0.00450477 |  | -0.0246081 | 10 | C | T | 0.279366 |
| ebi-a-GCST90092817 | Ratio of docosahexaenoic acid to total fatty acid levels | 65392056 | 3.5E-09 | 0.00445365 | 115006 | -0.0263023 | 10 | C | T | 0.279367 |
| met-d-M_VLDL_PL_pct | Phospholipids to total lipids ratio in medium VLDL | 65392056 | 3.7E-09 | 0.00442636 |  | -0.0245034 | 10 | C | T | 0.279366 |
| ebi-a-GCST90093039 | Triglycerides to total lipids ratio in very small VLDL | 65392056 | 4E-09 | 0.00436084 | 115082 | 0.0256677 | 10 | C | T | 0.27936 |
| met-d-M_VLDL_TG_pct | Triglycerides to total lipids ratio in medium VLDL | 65392056 | 4.3E-09 | 0.00435803 |  | 0.0237834 | 10 | C | T | 0.279366 |
| ukb-b-4711 | Usual walking pace | 65392056 | 4.4E-09 | 0.00141001 | 459915 | -0.0082748 | 10 | C | T | 0.280411 |
| met-d-IDL_TG_pct | Triglycerides to total lipids ratio in IDL | 65392056 | 5.8E-09 | 0.00445836 |  | 0.0237493 | 10 | C | T | 0.279366 |
| met-d-M_VLDL_FC_pct | Free cholesterol to total lipids ratio in medium VLDL | 65392056 | 7.5999E-09 | 0.00438749 |  | -0.0234229 | 10 | C | T | 0.279366 |
| ukb-b-14238 | Mineral and other dietary supplements: None of the above | 65392056 | 8.8E-09 | 0.00114262 | 461384 | 0.00657254 | 10 | C | T | 0.280408 |
| met-d-M_VLDL_C_pct | Cholesterol to total lipids ratio in medium VLDL | 65392056 | 1.1E-08 | 0.00434378 |  | -0.0231189 | 10 | C | T | 0.279366 |
| met-d-IDL_CE_pct | Cholesteryl esters to total lipids ratio in IDL | 65392056 | 1.1E-08 | 0.00452647 |  | -0.0240569 | 10 | C | T | 0.279366 |
| ebi-a-GCST90018959 | Height | 65392056 | 1.311E-08 | 0.0018 | 360388 | -0.0101 | 10 | C | T | 0.198736 |
| ebi-a-GCST90014017 | Diastolic blood pressure automated reading (UKB data field 4079) | 65392056 | 1.4296E-08 | 0.00242436 | 385801 | 0.0137457 | 10 | C | T |  |
| ebi-a-GCST90018962 | Lymphocyte count | 65392056 | 1.577E-08 | 0.0026 | 349856 | 0.0148 | 10 | C | T | 0.224876 |
| ukb-b-11535 | Mineral and other dietary supplements: Glucosamine | 65392056 | 1.7E-08 | 0.00091301 | 461384 | -0.005146 | 10 | C | T | 0.280408 |
| met-d-TG_by_PG | Ratio of triglycerides to phosphoglycerides | 65392056 | 1.9E-08 | 0.00428271 |  | 0.0221769 | 10 | C | T | 0.279371 |
| met-d-M_VLDL_CE_pct | Cholesteryl esters to total lipids ratio in medium VLDL | 65392056 | 1.9E-08 | 0.00433243 |  | -0.0227589 | 10 | C | T | 0.279366 |
| ieu-b-4869 | Bioavailable Testosterone | 65392056 | 2.2E-08 | 0.0036672 |  | 0.0205262 | 10 | C | T | 0.279871 |
| ebi-a-GCST004629 | Neutrophil count | 65392056 | 2.325E-08 | 0.00398865 | 170702 | 0.02228 | 10 | C | T | 0.2795 |
| ebi-a-GCST004613 | Sum neutrophil eosinophil counts | 65392056 | 2.398E-08 | 0.00399274 | 170384 | 0.0222816 | 10 | C | T | 0.2795 |
| ukb-d-30120_irnt | Lymphocyte count | 65392056 | 2.3985E-08 | 0.00264436 | 349856 | 0.0147572 | 10 | C | T | 0.281357 |
| ebi-a-GCST90002398 | Neutrophil count | 65392056 | 2.7E-08 | 0.00239058 | 408112 | 0.0132979 | 10 | C | T | 0.280864 |
| ebi-a-GCST90002401 | Platelet distribution width | 65392056 | 2.9E-08 | 0.00240386 | 408112 | 0.0133381 | 10 | C | T | 0.279762 |
| ebi-a-GCST90014015 | Urate levels (UKB data field 30880) | 65392056 | 3.2165E-08 | 0.00198159 | 389404 | 0.0109567 | 10 | C | T |  |
| ukb-d-30680_irnt | Calcium | 65392056 | 3.2878E-08 | 0.0027623 | 315153 | 0.015263 | 10 | C | T | 0.28119 |
| ukb-a-493 | Mineral and other dietary supplements: None of the above | 65392056 | 3.4533E-08 | 0.00134052 | 336314 | 0.0073955 | 10 | C | T | 0.280935 |
| met-d-S_VLDL_FC_pct | Free cholesterol to total lipids ratio in small VLDL | 65392056 | 3.5E-08 | 0.00440688 |  | -0.0224635 | 10 | C | T | 0.279366 |
| ebi-a-GCST90092925 | Phospholipids to total lipids ratio in medium VLDL | 65392056 | 3.8E-08 | 0.00442588 | 115082 | -0.0243445 | 10 | C | T | 0.27936 |
| ebi-a-GCST004614 | Granulocyte count | 65392056 | 3.97E-08 | 0.00399965 | 169822 | 0.0219667 | 10 | C | T | 0.2795 |
| met-d-S_VLDL_TG_pct | Triglycerides to total lipids ratio in small VLDL | 65392056 | 0.00000004 | 0.00446411 |  | 0.0231366 | 10 | C | T | 0.279366 |
| ebi-a-GCST004626 | Myeloid white cell count | 65392056 | 4.539E-08 | 0.00400868 | 169219 | 0.0219215 | 10 | C | T | 0.2795 |
| ebi-a-GCST004620 | Sum basophil neutrophil counts | 65392056 | 4.56E-08 | 0.00399631 | 170143 | 0.0218504 | 10 | C | T | 0.2795 |
| ebi-a-GCST90018951 | Calcium levels | 65392056 | 4.917E-08 | 0.0027 | 315153 | 0.0149 | 10 | C | T | 0.225805 |
| met-d-S_VLDL_C_pct | Cholesterol to total lipids ratio in small VLDL | 65392056 | 0.00000005 | 0.00448845 |  | -0.0231811 | 10 | C | T | 0.279366 |
| ukb-d-30680_raw | Calcium | 65392056 | 5.616E-08 | 0.00025926 | 315153 | 0.001408 | 10 | C | T | 0.28119 |
| ebi-a-GCST90012060 | Dickkopf-related protein 1 levels | 65392056 | 6.736E-08 | 0.0121 | 21758 | -0.0653 | 10 | C | T | 0.2565 |
| ebi-a-GCST90092927 | Triglycerides to total lipids ratio in medium VLDL | 65392056 | 7.5E-08 | 0.00434986 | 115082 | 0.023392 | 10 | C | T | 0.27936 |
| ukb-b-2148 | Facial ageing | 65392056 | 0.00000008 | 0.00118165 | 423999 | 0.00634324 | 10 | C | T | 0.280275 |
| met-d-DHA | Docosahexaenoic acid | 65392056 | 8.3E-08 | 0.00445736 |  | -0.0227803 | 10 | C | T | 0.279371 |
| met-d-S_VLDL_PL_pct | Phospholipids to total lipids ratio in small VLDL | 65392056 | 9.1E-08 | 0.0044158 |  | -0.0218677 | 10 | C | T | 0.279366 |
| ebi-a-GCST90092816 | Docosahexaenoic acid levels | 65392056 | 0.0000001 | 0.00437864 | 115006 | -0.0232981 | 10 | C | T | 0.279367 |
| met-d-M_LDL_FC_pct | Free cholesterol to total lipids ratio in medium LDL | 65392056 | 1.1E-07 | 0.00442122 |  | -0.0210723 | 10 | C | T | 0.279366 |
| met-d-PUFA_by_MUFA | Ratio of polyunsaturated fatty acids to monounsaturated fatty acids | 65392056 | 1.1E-07 | 0.00442319 |  | -0.0218948 | 10 | C | T | 0.279371 |
| ebi-a-GCST90092832 | Cholesterol to total lipids ratio in IDL | 65392056 | 1.1E-07 | 0.00447928 | 115082 | -0.0238094 | 10 | C | T | 0.27936 |
| ukb-a-389 | Standing height | 65392056 | 1.279E-07 | 0.00192337 | 336474 | -0.0101591 | 10 | C | T | 0.280935 |
| ebi-a-GCST90092921 | Free cholesterol to total lipids ratio in medium VLDL | 65392056 | 1.3E-07 | 0.0043875 | 115082 | -0.0231673 | 10 | C | T | 0.27936 |
| met-d-MUFA_pct | Ratio of monounsaturated fatty acids to total fatty acids | 65392056 | 1.4E-07 | 0.00442176 |  | 0.0212506 | 10 | C | T | 0.279371 |
| ebi-a-GCST90012010 | Proteinase-activated receptor 1 levels | 65392056 | 1.573E-07 | 0.0121 | 21758 | -0.0633 | 10 | C | T | 0.2564 |
| ebi-a-GCST90092917 | Cholesterol to total lipids ratio in medium VLDL | 65392056 | 0.00000017 | 0.00433425 | 115082 | -0.0226814 | 10 | C | T | 0.27936 |
| met-d-XS_VLDL_PL_pct | Phospholipids to total lipids ratio in very small VLDL | 65392056 | 0.0000002 | 0.00450093 |  | 0.0228682 | 10 | C | T | 0.279366 |
| ukb-a-494 | Mineral and other dietary supplements: Glucosamine | 65392056 | 2.1327E-07 | 0.00107572 | 336314 | -0.0055803 | 10 | C | T | 0.280935 |
| ieu-b-5117 | Adult BMI females | 65392056 | 2.2E-07 | 0.00219433 |  | 0.0113721 | 10 | C | T | 0.280317 |
| ebi-a-GCST90092842 | Triglycerides to total lipids ratio in IDL | 65392056 | 2.2E-07 | 0.00442374 | 115082 | 0.0229393 | 10 | C | T | 0.27936 |
| ieu-b-39 | diastolic blood pressure | 65392056 | 2.267E-07 | 0.0195 | 747735 | 0.1007 | 10 | C | T | 0.2747 |
| ukb-a-464 | Vitamin and mineral supplements: Multivitamins +/- minerals | 65392056 | 2.59E-07 | 0.00111287 | 335591 | -0.0057326 | 10 | C | T | 0.280935 |
| ebi-a-GCST90092834 | Cholesteryl esters to total lipids ratio in IDL | 65392056 | 2.6E-07 | 0.00448811 | 115082 | -0.0231248 | 10 | C | T | 0.27936 |
| ebi-a-GCST90092919 | Cholesteryl esters to total lipids ratio in medium VLDL | 65392056 | 2.6E-07 | 0.0043172 | 115082 | -0.0222452 | 10 | C | T | 0.27936 |
| ebi-a-GCST90000027 | Appendicular lean mass | 65392056 | 2.6E-07 | 0.00283572 | 244730 | -0.0137499 | 10 | C | T | 0.280576 |
| met-d-VLDL_size | Average diameter for VLDL particles | 65392056 | 3E-07 | 0.00430091 |  | 0.0202567 | 10 | C | T | 0.279366 |
| ukb-b-20531 | Leg fat percentage (right) | 65392056 | 0.00000032 | 0.00142165 | 454854 | 0.0072669 | 10 | C | T | 0.280501 |
| ukb-b-16812 | Vitamin and mineral supplements: Multivitamins +/- minerals | 65392056 | 3.5E-07 | 0.00095344 | 460351 | -0.0048591 | 10 | C | T | 0.280408 |
| met-d-S_VLDL_TG | Triglycerides in small VLDL | 65392056 | 3.5E-07 | 0.00447753 |  | 0.0213699 | 10 | C | T | 0.279366 |
| ebi-a-GCST90092983 | Ratio of triglycerides to phosphoglycerides | 65392056 | 3.5E-07 | 0.0042757 | 115006 | 0.0217806 | 10 | C | T | 0.279367 |
| ebi-a-GCST90092979 | Triglycerides to total lipids ratio in small VLDL | 65392056 | 3.5E-07 | 0.00444735 | 115082 | 0.0226627 | 10 | C | T | 0.27936 |
| ukb-b-18377 | Leg fat percentage (left) | 65392056 | 3.6E-07 | 0.00140338 | 454826 | 0.00713915 | 10 | C | T | 0.280506 |
| ebi-a-GCST90092969 | Cholesterol to total lipids ratio in small VLDL | 65392056 | 3.6E-07 | 0.00447497 | 115082 | -0.0227844 | 10 | C | T | 0.27936 |
| ieu-b-5118 | Adult BMI sex-combined | 65392056 | 4.2E-07 | 0.00152725 |  | 0.0077258 | 10 | C | T | 0.280457 |
| met-d-XL_VLDL_C_pct | Cholesterol to total lipids ratio in very large VLDL | 65392056 | 4.8E-07 | 0.00442274 |  | -0.0206076 | 10 | C | T | 0.279434 |
| met-d-XL_VLDL_CE_pct | Cholesteryl esters to total lipids ratio in very large VLDL | 65392056 | 4.9E-07 | 0.00442498 |  | -0.020553 | 10 | C | T | 0.279434 |
| ukb-b-16881 | Sitting height | 65392056 | 0.00000061 | 0.0016725 | 461536 | -0.0083424 | 10 | C | T | 0.280408 |
| ebi-a-GCST90092973 | Free cholesterol to total lipids ratio in small VLDL | 65392056 | 6.3E-07 | 0.0043831 | 115082 | -0.0218327 | 10 | C | T | 0.27936 |
| ebi-a-GCST90092934 | Ratio of omega-6 fatty acids to omega-3 fatty acids | 65392056 | 6.5E-07 | 0.00443661 | 115006 | 0.0220691 | 10 | C | T | 0.279367 |
| met-d-L_VLDL_CE_pct | Cholesteryl esters to total lipids ratio in large VLDL | 65392056 | 6.5999E-07 | 0.00441372 |  | -0.0206661 | 10 | C | T | 0.279362 |
| met-d-XL_VLDL_TG_pct | Triglycerides to total lipids ratio in very large VLDL | 65392056 | 6.8E-07 | 0.00447102 |  | 0.0204947 | 10 | C | T | 0.279434 |
| ebi-a-GCST90093037 | Phospholipids to total lipids ratio in very small VLDL | 65392056 | 6.8E-07 | 0.00449478 | 115082 | 0.022328 | 10 | C | T | 0.27936 |
| met-d-S_LDL_TG_pct | Triglycerides to total lipids ratio in small LDL | 65392056 | 7.3E-07 | 0.00447205 |  | 0.0199678 | 10 | C | T | 0.279366 |
| ukb-d-30600_irnt | Albumin | 65392056 | 7.5508E-07 | 0.0027417 | 315268 | 0.013562 | 10 | C | T | 0.28122 |
| ukb-b-11075 | Mineral and other dietary supplements: Fish oil (including cod liver oil) | 65392056 | 7.7999E-07 | 0.00107699 | 461384 | -0.0053214 | 10 | C | T | 0.280408 |
| ebi-a-GCST90018977 | Serum uric acid levels | 65392056 | 8.059E-07 | 0.0022 | 343836 | 0.011 | 10 | C | T | 0.209327 |
| ukb-b-2303 | Body mass index (BMI) | 65392056 | 8.7E-07 | 0.00221869 | 454884 | 0.010913 | 10 | C | T | 0.280499 |
| met-d-XL_VLDL_TG | Triglycerides in very large VLDL | 65392056 | 8.7E-07 | 0.00441304 |  | 0.0199614 | 10 | C | T | 0.279366 |
| ukb-b-5945 | Relative age of first facial hair | 65392056 | 9.2001E-07 | 0.0015142 | 204568 | -0.0074329 | 10 | C | T | 0.280616 |

| **Supplementary Table 10. OpenGWAS PheWAS results for SNP rs735465 for traits which met the predefined *P*-value threshold of < 9.99 x 10^-7^.** Columns are as reported by OpenGWAS, where: ID = OpenGWAS GWAS ID, Trait = name of trait (may be Ensembl ID for gene expression), Position = genomic position, P = *P*-value of SNP-trait association, SE = standard error, N = sample size (where given on OpenGWAS), Beta = beta of SNP-trait association, CHR = chromosome, EA = effect alelle, NEA = other allele, EAF = effect allele frequency (where available). | | | | | | | | | | |
| --- | --- | --- | --- | --- | --- | --- | --- | --- | --- | --- |
| **ID** | **Trait** | **Position** | **P** | **SE** | **N** | **Beta** | **CHR** | **EA** | **NEA** | **EAF** |
| eqtl-a-ENSG00000110906 | ENSG00000110906 | 110136274 | 3.7196E-55 | 0.0137239 | 31346 | -0.214682 | 12 | G | C | 0.755205 |
| eqtl-a-ENSG00000076555 | ENSG00000076555 | 110136274 | 2.6687E-19 | 0.013803 | 31346 | 0.123973 | 12 | G | C | 0.755205 |
| eqtl-a-ENSG00000111231 | ENSG00000111231 | 110136274 | 1.062E-09 | 0.0138242 | 25858 | -0.0843275 | 12 | G | C | 0.755205 |
| ukb-b-11535 | Mineral and other dietary supplements: Glucosamine | 110136274 | 1.3E-08 | 0.0009649 | 461384 | 0.0054867 | 12 | G | C | 0.761171 |
| eqtl-a-ENSG00000151148 | ENSG00000151148 | 110136274 | 9.0317E-08 | 0.0138284 | 31346 | 0.0739142 | 12 | G | C | 0.755205 |
| ebi-a-GCST90018734 | Gamma glutamyl transpeptidase | 110136274 | 1.3E-07 | 0.00378437 | 133471 | 0.0199913 | 12 | G | C | 0.524945 |
| eqtl-a-ENSG00000139433 | ENSG00000139433 | 110136274 | 3.8063E-07 | 0.0138298 | 13925 | -0.0702344 | 12 | G | C | 0.755205 |
