# Supplementary material for: Association between glucosamine use and albuminuria in the UK: a cohort and Mendelian randomisation study
Source: BMJ Open. 2025 Nov 21;15(11):e096344. doi: 10.1136/bmjopen-2024-096344 (PMC12658525; doi:10.1136/bmjopen-2024-096344)
Supplement: Supplementary file 2 [file bmjopen-2024-096344.reviewer_comments.pdf]

PEER REVIEW HISTORY

BMJ Open publishes all reviews undertaken for accepted manuscripts. Reviewers are asked to complete a checklist review form and are provided with free text boxes to elaborate on their assessment. These free text comments are reproduced below.

ARTICLE DETAILS

Title (Provisional)

Association between glucosamine use and albuminuria in the UK: a cohort and Mendelian Randomisation study

Authors

Hayward, Samantha Jane Lucy; Constantinescu, Andrei; Hazelwood, Emma; Butler, Matthew J; Satchell, Simon C; Vincent, Emma E

VERSION 1 - REVIEW

**Reviewer**	**1**
**Name**	**Timmins, Iain R.**
**Affiliation**	**AstraZeneca PLC, Oncology R&D**
**Date**	**07-Jul-2025**
**COI**	**None**

Statistical Review: This is a well-conducted and comprehensive analysis of glucosamine use on albuminuria with observational and MR analyses. I appreciate the issue of ‘supplemented glucosamine’ rather than ‘directly measured glucosamine’ raises challenges for performing these analyses. Here are comments on the statistical aspects of study design – in particular, the observational associations and the issue of confounding, MR study design, and Phenome-wide association study design, and its interpretation and presentation. Hopefully these comments may be helpful for the authors.

Confounding.

1. In the observational analyses, the authors have rightly adjusted for confounders using multivariate methods. Sex is one potential confounder that could be included – since supplement use is higher for women, and albuminuria risk is likely higher for men (due to possible hormonal factors, cardiovascular risk profiles, and lifestyle behaviours). Ethnicity may be another such factor, since this could impact rates of supplement use and outcome risk. Physical activity could also be considered, and also the use of other supplements/medications, and dietary factors – to take into account healthy lifestyle choices. Hypertension/blood pressure could also be a confounder. Hence it may be worth

looking at further models with greater levels of covariate adjustment than currently performed.

2. The authors state that “an alternative explanation is that the observed relationships between glucosamine use and positive health outcomes may be a result of unmeasured or residual confounding” and “residual confounding may still be at play”. I accept that this kind of statement is common in epidemiology studies (and I have written such things myself) – however it is worth noting that there are statistical techniques to quantify the minimum strength of association of an unmeasured confounder that would nullify the observed associations, such as using E-values/tipping point analysis, so potentially more could be done to investigate this issue.

3. There is the issue of supplement use being a health-seeking behaviour that confounds results – as currently mentioned “people who take glucosamine may be more engaged with their health, have certain healthy behaviours or take other supplementary treatments which have had a positive effect on albuminuria”. To additionally address this, negative control exposures could be considered – for instance, what supplements are recorded in UK Biobank that do not plausibly impact albuminuria risk, and what is the evidence of association with albuminuria for these exposures - would that help quantify the potential effect of supplementation use as a health-seeking behaviour?

Mendelian randomisation study

1. Using IVW, MR Egger and Weighted median with genome-wide significant SNPs is a good strategy, though given there’s only 5 SNPs, these analyses may be underpowered (a power calculation could be performed to formally assess?). Hence other methods might be worth exploring to boost power. One could look at using MR-RAPS (doi.org/10.1214/19-AOS1866) while varying the P-value threshold of SNPs to P < 10^-6, 10^-5 and 10^-4 to include more SNPs, while reporting median F-statistic. That is an approach that provides good estimation properties for instrumental variable analysis with less stringent P-value thresholds. Another approach that improves power by incorporating all variants is MR-CAUSE (doi.org/10.1038/s41588-020-0631-4). There may be other available methods besides these.

2. I would add confounders to the DAG in Figure 3B.

Phenome-wide association

1. Using Bonferroni correction of P/50,044 is perhaps conservative given there will be a lot of correlation across these 50,044 phenotypes. If there’s a straightforward approaches to identify the number of independent phenotypes among these (perhaps this has been calculated by other authors somewhere), they may be worth exploring. But this may well not be easily possible.

2. In motivating this analysis we read that “In order to demonstrate the issues in using liability to glucosamine use, rather than measured glucosamine levels, we performed a phenome-wide association analysis…”. Though how do we know we wouldn’t encounter the

same issues for measured glucosamine levels, since the SNPs for that phenotype might also demonstrate widespread pleiotropy? This analysis in itself doesn't provide the comparison with measured glucosamine levels, so we can't necessarily say these conclusions are specific to just supplemented glucosamine use. But perhaps we can hypothesise that measured glucosamine levels would provide more credible and biologically precise SNPs as instruments? Is there anything more that can be said about the 5 SNPs beyond their statistical significance (P<5*10^-8) - what are their biological functions/annotations, what are the relevant genes, have existing GWAS analyses explored this at all?

3. It may be worth considering figure designs to better communicate the findings here, since at present we have to look through several supplementary tables. For PheWAS, we sometimes see heatmaps of Z-scores (Figure 2 of doi:10.1038/s41467-018-06540-3) or the use of Manhattan plots (Figure 1 of doi:10.1038/s41467-020-16022-0) perhaps grouping phenotypes into broader categories of traits.

Many thanks for the opportunity to review, and best wishes to the authors on the publication of their findings.

**Reviewer**	**2**
**Name**	**Grant, Andrew J**
**Affiliation**	**University of Sydney CAR**
**Date**	**12-Aug-2025**
**COI**	**None**

The paper considers the association between glucosamine and albuminuria using observational evidence and then using Mendelian randomization to try and estimate causal effects. Overall, it is a well-written paper, and the statistical analyses have been appropriately implemented and presented. I have the following comments in relation to the statistical aspects of the work.

- The main analyses use ordinal logistic regression with the outcome categorised according to conventional thresholds. Could some comment be given on the proportional odds assumption used in these models? It would be good to have discussion of the plausibility of this assumption in light of the chosen outcome categories, potentially with statistical tests for the assumption as well.

- In describing the datasets from which the GWAS summary statistics were sourced, a reference to the underlying GWAS for the exposure associations could be included in addition to the MRC-IEU database reference.

- The genetic instruments were chosen using statistical methods, but it would be good to give some comment on any biological link between the chosen SNPs and the exposure.

- The Mendelian randomization study used a variety of estimation methods, including MR-Egger, weighted median, and weighted mode. It would be good to briefly describe the assumptions of these approaches, and to explain why these particular methods were chosen as sensitivity analyses. Furthermore, Table 3 does not include the weighted mode results.

- The chosen genetic instruments included only 5 SNPs with very low R-squared values. This would mean that the Mendelian randomization study is low powered, evidenced by the very wide confidence intervals for the estimates. A suggestion would be to increase the p-value threshold, which may increase the number of genetic instruments and therefore the study's power. This could allow for more insight to be gained from this part of the analysis.

VERSION 1 - AUTHOR RESPONSE

Reviewer: 1 - Dr. Iain R. Timmins, AstraZeneca PLC

This is a well-conducted and comprehensive analysis of glucosamine use on albuminuria with observational and MR analyses. I appreciate the issue of 'supplemented glucosamine' rather than 'directly measured glucosamine' raises challenges for performing these analyses. Here are comments on the statistical aspects of study design – in particular, the observational associations and the issue of confounding, MR study design, and Phenome-wide association study design, and its interpretation and presentation. Hopefully these comments may be helpful for the authors.

1) Confounding:

1.1) In the observational analyses, the authors have rightly adjusted for confounders using multivariate methods. Sex is one potential confounder that could be included – since supplement use is higher for women, and albuminuria risk is likely higher for men (due to possible hormonal factors, cardiovascular risk profiles, and lifestyle behaviours). Ethnicity may be another such factor, since this could impact rates of supplement use and outcome risk. Physical activity could also be considered, and also the use of other supplements/medications, and dietary factors – to take into account healthy lifestyle choices. Hypertension/blood pressure could also be a confounder. Hence it may be worth looking at further models with greater levels of covariate adjustment than currently performed.

We thank the reviewer for drawing attention to these additional factors that may act as confounders. We have carefully considered each of these:

We agree that sex may be confounding the analysis as there is good evidence that glucosamine use is higher in women (PMIDs: 22859995 and 24219123) and sex differences in albuminuria have also been demonstrated previously. (DOI:10.1681/ASN.V1341034). Therefore, we have added sex in as a co-variate in all the multivariable models (main, subgroup and sensitivity analyses); the results are consistent with those that were submitted in the original manuscript and are presented in revised versions of Table 2, Figure 2 and Supplementary Tables 4-5. We also agree that blood pressure is a potential confounding factor and we have included a sensitivity

analysis with blood pressure as a co-variate. The results from this sensitivity analysis are presented in Supplementary Table 4 and are similar to those from the main analysis. The methods section has been updated to reflect these changes in co-variates (line 156–163).

With regard to physical activity, it is well-established that transient albuminuria can occur after extremely intense exercise (PMID: 18089464). However, it is unlikely that a substantial proportion of participants engaged in extremely intense exercise immediately prior to sample collection, and therefore any impact on the results is expected to be minimal. Outside of the scenario of extreme intense exercise, general physical activity is likely to be associated with glucosamine use and albuminuria, but through its relationship with obesity. Therefore, we believe that physical activity is an upstream factor to obesity, with obesity being the true confounder. As such, we have adjusted the main analysis for measures of obesity (BMI and waist-to-hip ratio).

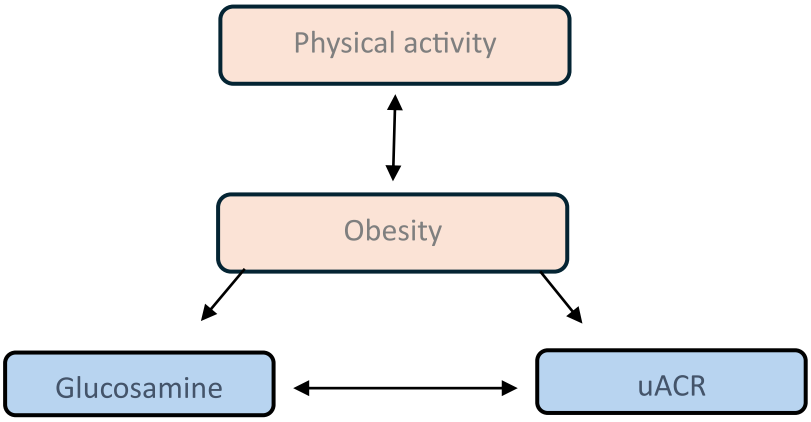

An association between ethnicity and glucosamine use has previously been demonstrated (PMIDs:35794906), with higher use shown in non-Hispanic white people. However, in clinical practice it is not generally accepted that uACR levels differ by ethnicity, with the same reference ranges and risk categories used across all ethnic groups in international guidance (Kidney Disease Improving Global Outcomes guidance 2024 - https://kdigo.org/wp-content/uploads/2024/03/KDIGO-2024-CKD-Guideline.pdf). Therefore, after careful consideration, we do not feel that ethnicity fulfils the criteria of a confounding factor (doi.org/10.1038/sj.ki.5002650) and so have not included it as a co-variate in the models.

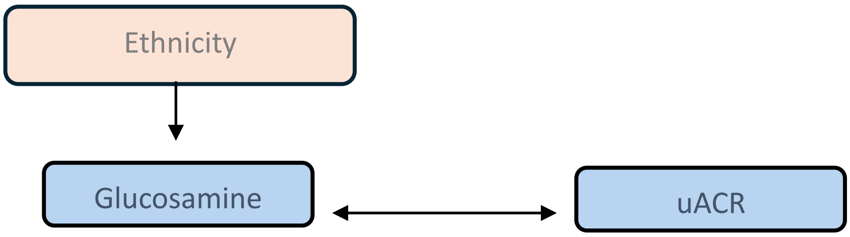

1.2) The authors state that "an alternative explanation is that the observed relationships between glucosamine use and positive health outcomes may be a result of unmeasured or residual confounding" and "residual confounding may still be at play". I accept that this kind of statement is common in epidemiology studies (and I have

written such things myself) – however it is worth noting that there are statistical techniques to quantify the minimum strength of association of an unmeasured confounder that would nullify the observed associations, such as using E-values/tipping point analysis, so potentially more could be done to investigate this issue.

Thank you for highlighting these methods to quantify the presence of unmeasured confounding, we have looked into them in detail and weighed up their benefits and limitations. Overall, we have reservations in reporting an E-value in our study as there is little consensus in the literature or guidance as to what indicates a high or low enough value to draw a meaningful conclusion about residual confounding (doi.org/10.1093/ije/dyz261 and doi.org/10.7326/M18-2159). We have added this information into the discussion section of the manuscript (line 378-381).

1.3) There is the issue of supplement use being a health-seeking behaviour that confounds results – as currently mentioned "people who take glucosamine may be more engaged with their health, have certain healthy behaviours or take other supplementary treatments which have had a positive effect on albuminuria". To additionally address this, negative control exposures could be considered – for instance, what supplements are recorded in UK Biobank that do not plausibly impact albuminuria risk, and what is the evidence of association with albuminuria for these exposures - would that help quantify the potential effect of supplementation use as a health-seeking behaviour?

This is an interesting idea, which we were keen to pursue. We have spent some time reviewing the data available in UK biobank to determine whether it could be investigated and included in the manuscript. UK biobank participants were specifically asked about their use of fish oil, iron, calcium and zinc supplements, as well as glucosamine. Previous studies have suggested an association between fish oil and albuminuria (summarised in this review, PMID: 28710179). In addition, lower zinc levels have shown to associate with albuminuria (doi.org/10.1016/j.jtemb.2021.126755 and 10.1186/s12882-022-03040-x) and zinc supplementation has been reported to reduce albuminuria in a randomised control trial (PMID: 18795212). Iron and calcium supplements are part of routine treatment for chronic kidney disease, which is in part defined by albuminuria (Kidney Disease Improving Global Outcomes guidance 2024 - https://kdigo.org/wp-content/uploads/2024/03/KDIGO-2024-CKD-Guideline.pdf). Therefore, unfortunately, evaluating the association between other supplement use and albuminuria in UKBB is unlikely to help quantify health-seeking behaviour, due to their own known independent associations with albuminuria.

2) Mendelian randomisation study

2.1) Using IVW, MR Egger and Weighted median with genome-wide significant SNPs is a good strategy, though given there's only 5 SNPs, these analyses may be underpowered (a power calculation could be performed to formally assess?). Hence other methods might be worth exploring to boost power. One could look at using MR-RAPS (doi.org/10.1214/19-AOS1866) while varying the P-value threshold of SNPs to P < 10^-6,

10^-5 and 10^-4 to include more SNPs, while reporting median F-statistic. That is an approach that provides good estimation properties for instrumental variable analysis with less stringent P-value thresholds. Another approach that improves power by incorporating all variants is MR-CAUSE (doi.org/10.1038/s41588-020-0631-4). There may be other available methods besides these.

Thank you for this suggestion, we agree that power is an important consideration in Mendelian randomisation (MR) studies and that an instrument containing only 5 SNPs may be underpowered. However, we are concerned that relaxing the genome-wide significance threshold for instrument selection would lead to weak instrument bias . As noted elsewhere (e.g. doi.org/10.1101/2025.06.03.25328787), the use of SNPs selected at less stringent thresholds (e.g. $p < 10^{-5}$ or 10^{-6}) introduces substantial risk of violating core MR assumptions, particularly the relevance and exclusion restriction assumptions, and increases susceptibility to type I error. While this practice is common, we feel it undermines the validity of the causal inference and therefore we have restricted our analyses to genome-wide significant SNPs. However, we have highlighted the concern over power and the number of SNPs in the instrument in the discussion section of the paper (line 385-387).

In terms of methodology, we recognise that there are many MR approaches available – Table 1 of a recent primer (https://www.nature.com/articles/s43586-022-00099-6) lists more than 30 methods, and this is not exhaustive. Our primary analyses employed IVW, MR-Egger, weighted median, and weighted mode estimators, which represent a balance of complementary assumptions. We have added additional text to the methods section of the manuscript (line 202-219) to explain the assumptions of each of these methods and highlight the complementary advantages and disadvantages of each. All of our instruments had F-statistics above 10, suggesting that weak instrument bias is unlikely to be a major concern. For this reason, methods specifically designed to address weak instruments, such as MR-RAPS, are less applicable here. Similarly, while approaches such as MR-CAUSE incorporate all variants to improve power, their reliance on less stringent thresholds raises the same concerns about weak instrument bias as noted above.

2.2) I would add confounders to the DAG in Figure 3B.

Thank you - we have added the confounder of obesity into the DAG in Figure 3B.

3) Phenome-wide association

3.1) Using Bonferroni correction of P/50,044 is perhaps conservative given there will be a lot of correlation across these 50,044 phenotypes. If there's a straightforward approaches to identify the number of independent phenotypes among these (perhaps this has been calculated by other authors somewhere), they may be worth exploring. But this may well not be easily possible.

We agree with the reviewer that the Bonferroni correction is likely overly-conservative given the probable correlation between certain phenotypes; this a common problem in PheWAS studies. Despite this, the Bonferroni-corrected p-value threshold still remains the most widely used p-value threshold in PheWAS analyses, as there is no clear consensus on how to best define the number of independent phenotypes. We agree that determining a more appropriate p-value threshold to use in PheWAS studies would be of benefit, however, this falls outside of the remit of this current study.

*3.2) In motivating this analysis we read that "In order to demonstrate the issues in using liability to glucosamine use, rather than measured glucosamine levels, we performed a phenome-wide association analysis…". Though how do we know we wouldn't encounter the same issues for measured glucosamine levels, since the SNPs for that phenotype might also demonstrate widespread pleiotropy? This analysis in itself doesn't provide the comparison with measured glucosamine levels, so we can't necessarily say these conclusions are specific to just supplemented glucosamine use. But perhaps we can hypothesise that measured glucosamine levels would provide more credible and biologically precise SNPs as instruments? Is there anything more that can be said about the 5 SNPs beyond their statistical significance (P<5*10^-8) - what are their biological functions/annotations, what are the relevant genes, have existing GWAS analyses explored this at all?*

We thank the reviewer for highlighting that SNPs which are associated with measured glucosamine levels may also demonstrate horizontal pleiotropy and we have added text to the discussion to reflect this (line 352-354). We agree with this point, however, we would expect the horizontal pleiotropy to be reduced as glucosamine levels are a much more specific trait than glucosamine use. Unfortunately, in the absence of a GWAS of glucosamine levels we are unable to prove this.

We agree that more information about the 5 SNPs contained in the glucosamine instrument would be a good addition to the manuscript. We have added the SNP annotations into the MR analyses results section (line 269-272). We have also searched the literature for any plausible published biological link between these SNPs and glucosamine synthesis, metabolism or any known disease – no links were identified. We have added text to the discussion section of the manuscript to highlight this to the reader (line 348-349).

3.3) It may be worth considering figure designs to better communicate the findings here, since at present we have to look through several supplementary tables. For PheWAS, we sometimes see heatmaps of Z-scores (Figure 2 of doi:10.1038/s41467-018-06540-3) or the use of Manhattan plots (Figure 1 of doi:10.1038/s41467-020-16022-0) perhaps grouping phenotypes into broader categories of traits. Many thanks for the opportunity to review, and best wishes to the authors on the publication of their findings.

Thank you for this suggestion. We performed the PheWAS to show the number of phenotypes that are associated with the instrument SNPs (>200) and illustrate that horizontal pleiotropy may be affecting the MR. The PheWAS findings support our argument that instrumenting glucosamine use is problematic and the results from our study (and others, which have not explicitly highlighted this issue to the reader,) should be interpreted within this limitation. As we wish to highlight the number of traits, rather than the specific traits, identified in the PheWAS we have kept the findings in tables. However, if the reviewer feels strongly that a figure is a better way to communicate these findings, we would happily add in additional supplementary figures as well.

Reviewer: 2 - Dr. Andrew J Grant, University of Sydney CAR

The paper considers the association between glucosamine and albuminuria using observational evidence and then using Mendelian randomization to try and estimate causal effects. Overall, it is a well-written paper, and the statistical analyses have been appropriately implemented and presented. I have the following comments in relation to the statistical aspects of the work.

1) The main analyses use ordinal logistic regression with the outcome categorised according to conventional thresholds. Could some comment be given on the proportional odds assumption used in these models? It would be good to have discussion of the plausibility of this assumption in light of the chosen outcome categories, potentially with statistical tests for the assumption as well.

Thank you to the reviewer for this suggestion. We have added text into the methods explaining the proportional odds assumption in ordinal logistic regression (line 156-160). We have also made it clearer that the two sensitivity analyses which used linear regression (with albuminuria as a continuous variable) were included in case this assumption was violated (line 156-160). Reassuringly, the results from the ordinal logistic regression and linear regression analyses were consistent (Table 2 and Supplementary Table 3). We decided to keep the ordinal logistic regression model as the primary analysis as the results would be easy to interpret in relation to albuminuria thresholds used in clinical practice.

2) In describing the datasets from which the GWAS summary statistics were sourced, a reference to the underlying GWAS for the exposure associations could be included in addition to the MRC-IEU database reference.

Thank you for bringing this to our attention. We have added the MRC-IEU OpenGWAS database reference number for the UK biobank glucosamine use GWAS to the manuscript (line 184).

3) The genetic instruments were chosen using statistical methods, but it would be good

to give some comment on any biological link between the chosen SNPs and the exposure.

We agree with that this would strengthen the manuscript. We have searched the published literature to try and identify any plausible biological links between the 5 SNPs contained in the glucosamine instrument and glucosamine synthesis or metabolism – we have not identified any links. We have added text to the discussion section of the manuscript to reflect this and highlight it to the reader (line 348-349). We have also added the gene annotation for the 5 SNPs into results section of the manuscript (line 269-272).

4) The Mendelian randomization study used a variety of estimation methods, including MR-Egger, weighted median, and weighted mode. It would be good to briefly describe the assumptions of these approaches, and to explain why these particular methods were chosen as sensitivity analyses. Furthermore, Table 3 does not include the weighted mode results.

Our primary analyses employed IVW, with MR-Egger, weighted median, and weighted mode estimators as sensitivity analyses. The inclusion of these methods provides robustness to the results as they have different and complementary assumptions. The text given below has been added to the methods section of the manuscript to provide further detail of these methods and their assumptions (line 202-219). In addition, thank you for highlighting that Table 3 does not include the weighted mode results from the MR analyses, this has been amended and these results are now included in this table.

'Inverse-variance weighting (IVW) was used for the main effect estimate of genetically predicted glucosamine use on uACR. IVW assumes that all the SNPs used in the genetic instrument are valid[1]. Therefore, the inclusion of pleiotropic variants in an IVW analysis can lead to biased causal effect estimates[2]. To address this assumption and evaluate the possibility of horizontal pleiotropy, sensitivity analyses using MR-Egger, weighted median and mode were performed; these methods can offer more consistent causal effect estimates when the IV assumptions are violated but have different underlying assumptions. MR-Egger can give consistent estimates when 100% of the genetic variants are invalid IVs. However, MR-Egger requires that the pleiotropic effects of the IV SNPs on the outcome are independent of their associations with the exposure (Instrument Strength Independent of Direct Effects – InSIDE – assumption)[3]. Both weighted median and weighted mode approaches can produce more consistent causal estimates regardless of the type of horizontal pleiotropy (ie. the InSIDE assumption can be violated)[3]. Weighted median can provide consistent estimates when less than 50% of the IVs are invalid, whereas weighted mode can perform well in the context of 50-100% invalid IVs[3]. A weighted mode approach carries the additional assumption that the largest number of similar SNP-specific causal estimates come from the valid IVs (Zero Modal Pleiotropy Assumption – ZEMPA)[3]'

1. Bowden J, Davey Smith G, Haycock PC, Burgess S. Consistent Estimation in

Mendelian Randomization with Some Invalid Instruments Using a Weighted Median Estimator. Genet Epidemiol. 2016 May;40(4):304–14.
2. Burgess S, Thompson SG. Use of allele scores as instrumental variables for Mendelian randomization. Int J Epidemiol. 2013 Aug;42(4):1134–44.
3. Hartwig FP, Davey Smith G, Bowden J. Robust inference in summary data Mendelian randomization via the zero modal pleiotropy assumption. Int J Epidemiol. 2017 Dec 1;46(6):1985–98.

5) The chosen genetic instruments included only 5 SNPs with very low R-squared values. This would mean that the Mendelian randomization study is low powered, evidenced by the very wide confidence intervals for the estimates. A suggestion would be to increase the p-value threshold, which may increase the number of genetic instruments and therefore the study's power. This could allow for more insight to be gained from this part of the analysis.

Thank you for this comment, please see our response to reviewer 1 point 2.1 who had proposed the same suggestion.

VERSION 2 - REVIEW

**Reviewer**	**1**
**Name**	**Timmins, Iain R.**
**Affiliation**	**AstraZeneca PLC, Oncology R&D**
**Date**	**10-Oct-2025**
**COI**	

Thank you to the Author's for their considered and detailed reply to the comments raised. The manuscript is comprehensive in characterising glucosamine use and albuminuria.

The consideration of confounders based on clinical insights in the Author's reply was a valuable addition, and was also very interesting to read. Likewise the additional detail on MR methods was helpful and strengthens the motivation for these analyses/sensitivity analyses. The difficulties in gaining clear biological insights through the annotations of SNPs also interesting to note, and remains a challenge in any Mendelian randomisation for complex traits. The concerns regarding potential weak instrument bias of lowering the P-value threshold for SNPs are understandable and I accept the arguments made.

Thank you for the interesting comment and exploration of negative control experiments in the response, which was also illuminating.

Regarding PheWas and all other features of the study, I would suggest no further analyses, tables or figures are required. Best wishes to the authors on the publication of their findings.

Minor.

I would use the term "waist-to-hip ratio" throughout rather than "waist to hip ratio" as I think using a hyphen is the more accepted form (on the basis that it's a compound modifier).

I would avoid capitalising the "R" when using variant names, even at the start of a sentence, like e.g. Rs1466978, since this is a standard label name. It is I believe acceptable to begin a sentence with "rs1466978", but if this is not preferable for whatever reason, we can always start it with "SNP rs1466978... " or rephrase the sentence to avoid starting with rs.

Personally, writing for a British journal, I would follow the British English spelling of "randomisation" throughout rather than the "z".

I would go for e.g. "First", "Second", .. "Sixth" over "Sixthly" when enumerating/listing several points for scientific journal writing, I think that would be more standard.

**Reviewer**	**2**
**Name**	**Grant, Andrew J**
**Affiliation**	**University of Sydney CAR**
**Date**	**02-Oct-2025**
**COI**	

I thank the authors for their responses to my comments, which have been satisfactorily addressed. I have no further comments on the revised manuscript.

VERSION 2 - AUTHOR RESPONSE

Reviewer: 1

Dr. Iain R. Timmins, AstraZeneca PLC
Comments to the Author:
Thank you to the Author's for their considered and detailed reply to the comments raised. The manuscript is comprehensive in characterising glucosamine use and albuminuria. The consideration of confounders based on clinical insights in the Author's reply was a valuable addition, and was also very interesting to read. Likewise the additional detail on MR methods was helpful and strengthens the motivation for these analyses/sensitivity analyses. The difficulties in gaining clear biological insights through the annotations of SNPs also interesting to note, and remains a challenge in any Mendelian randomisation for complex traits. The concerns regarding potential weak instrument bias of lowering the P-value threshold for SNPs are understandable and I accept the arguments made.Thank you for the interesting comment and exploration of negative control experiments in the response, which was also illuminating.Regarding PheWas and all other features of the study, I would suggest no further analyses, tables

or figures are required. Best wishes to the authors on the publication of their findings.

Minor.
I would use the term "waist-to-hip ratio" throughout rather than "waist to hip ratio" as I think using a hyphen is the more accepted form (on the basis that it's a compound modifier).

Thank you, we have added hyphens as you have suggested.

I would avoid capitalising the "R" when using variant names, even at the start of a sentence, like e.g. Rs1466978, since this is a standard label name. It is I believe acceptable to begin a sentence with "rs1466978", but if this is not preferable for whatever reason, we can always start it with "SNP rs1466978... " or rephrase the sentence to avoid starting with rs.

We have made this amendment to the SNP names.

Personally, writing for a British journal, I would follow the British English spelling of "randomisation" throughout rather than the "z".

We have changed the spelling to the British English version.

I would go for e.g. "First", "Second", .. "Sixth" over "Sixthly" when enumerating/listing several points for scientific journal writing, I think that would be more standard.

Thank you, we have altered the text in the discussion as you have suggested.

Reviewer: 2
Dr. Andrew J Grant, University of Sydney CAR
Comments to the Author:
I thank the authors for their responses to my comments, which have been satisfactorily addressed. I have no further comments on the revised manuscript.
